# Microarray assessment of N-glycan-specific IgE and IgG profiles associated with *Schistosoma mansoni* infection in rural and urban Uganda

**DOI:** 10.1038/s41598-019-40009-7

**Published:** 2019-03-05

**Authors:** Gyaviira Nkurunungi, Angela van Diepen, Jacent Nassuuna, Richard E. Sanya, Margaret Nampijja, Irene Nambuya, Joyce Kabagenyi, Sonia Serna, Niels-Christian Reichardt, Ronald van Ree, Emily L. Webb, Alison M. Elliott, Maria Yazdanbakhsh, Cornelis H. Hokke

**Affiliations:** 1 Immunomodulation and Vaccines Programme, Medical Research Council/Uganda Virus Research Institute and London School of Hygiene and Tropical Medicine (MRC/UVRI and LSHTM) Uganda Research Unit, Entebbe, Uganda; 2 Department of Clinical Research, London School of Hygiene and Tropical Medicine, London, United Kingdom; 3 Department of Parasitology, Leiden University Medical Center, Leiden, The Netherlands; 4 College of Health Sciences, Makerere University, Kampala, Uganda; 5 Glycotechnology Laboratory, Centro de Investigación Cooperativa en Biomateriales (CIC biomaGUNE), San Sebastián, Spain; 6 Centro de Investigación Biomédica en Red en Bioingeniería, Biomateriales y Nanomedicina (CIBER-BBN), San Sebastián, Spain; 7 Amsterdam University Medical Centers, Departments of Experimental Immunology and of Otorhinolaryngology, Amsterdam, The Netherlands; 8 MRC Tropical Epidemiology Group, Department of Infectious Disease Epidemiology, London School of Hygiene and Tropical Medicine, London, United Kingdom

## Abstract

Core β-1,2-xylose and α-1,3-fucose are antigenic motifs on schistosome N-glycans, as well as prominent IgE targets on some plant and insect glycoproteins. To map the association of schistosome infection with responses to these motifs, we assessed plasma IgE and IgG reactivity using microarray technology among Ugandans from rural *Schistosoma mansoni* (*Sm*)-endemic islands (n = 209), and from proximate urban communities with lower *Sm* exposure (n = 62). IgE and IgG responses to core β-1,2-xylose and α-1,3-fucose modified N-glycans were higher in rural versus urban participants. Among rural participants, IgE and IgG to core β-1,2-xylose were positively associated with *Sm* infection and concentration peaks coincided with the infection intensity peak in early adolescence. Responses to core α-1,3-fucose were elevated regardless of *Sm* infection status and peaked before the infection peak. Among urban participants, *Sm* infection intensity was predominantly light and positively associated with responses to both motifs. Principal component and hierarchical cluster analysis reduced the data to a set of variables that captured core β-1,2-xylose- and α-1,3-fucose-specific responses, and confirmed associations with *Sm* and the rural environment. Responses to core β-1,2-xylose and α-1,3-fucose have distinctive relationships with *Sm* infection and intensity that should further be explored for associations with protective immunity, and cross-reactivity with other exposures.

## Introduction

Schistosomiasis is second only to malaria as a parasitic cause of human morbidity, with over 230 million infections globally, the majority of which occur in tropical and subtropical sub-Saharan Africa^1–3^. Despite important strides in coverage of anthelminthic treatment, reductions in infection prevalence have only been modest^4–6^, and the long struggle for a vaccine breakthrough continues^7^. The host immunological response to *Schistosoma* infection is shaped to a significant extent by schistosome surface-exposed and secreted glycans and glycoproteins. For example, anti-glycan antibody responses dominate the host humoral response to schistosome larvae and eggs^8–10^ and *Schistosoma* soluble egg antigen (SEA)-mediated Th2-polarisation profoundly relies on glycosylation^11,12^. In a mouse model for periovular granuloma formation, periodate treatment of SEA-coated beads inhibited their granulomogenic activity^13^, further demonstrating the functional relevance of glycan-specific responses in *Schistosoma*-mediated immunity and pathology. A better understanding of the human immune response to the *Schistosoma* glycome may be beneficial to the current drive towards identification of better *Schistosoma* diagnostic markers and potent vaccine candidates^14–18^.

**Figure 1:**
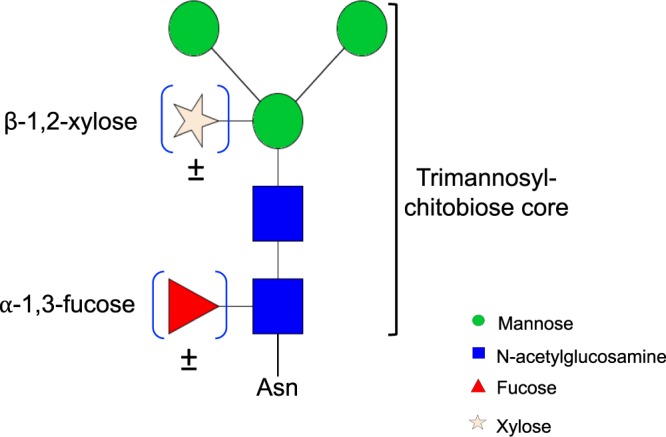
Non-mammalian carbohydrate substitutions on the N-glycan core. Non-mammalian monosaccharide substitutions are denoted by blue brackets. ±implies that motifs in brackets are present or absent in different species. Figure drawn using GlycoWorkbench software, version 2.1 (European Carbohydrates Database Project).

Current insights into the *Schistosoma* glycome, the most characterised among parasites, have been particularly aided by mass spectrometry-based (MS) studies^19–21^. Analysis of asparagine (N)-linked glycans expressed by schistosomes reveals two standout, non-mammalian substitutions^22,23^ on the trimannosyl-chitobiose core (Man_3_GlcNAc_2_, conserved in all eukaryotes): an α-1,3-fucose (α3Fuc) linked to the asparagine-linked N-acetylglucosamine (GlcNAc) of the chitobiose component and a β-1,2-xylose (β2Xyl) linked to the β-mannose of the trimannosyl component^24^ (Fig. 1). These substitutions are also found on nematode glycans from *Haemonchus contortus* and *Caenorhabditis elegans*^25–28^, and on invertebrate^29,30^ and plant glycans^31–33^, but have so far not been detected on glycans from other helminths prevalent in the tropics^19^. Detailed MS studies have neither detected core β2Xyl nor core α3Fuc modified N-glycans in adult schistosome worms but both are present in miracidia and eggs, while cercariae express core β2Xyl but no α3Fuc on the core GlcNAc^19^. Other common alterations to the schistosome Man_3_GlcNAc_2_ core include addition of antennae composed of GalNAcβ1-4GlcNAc (LacdiNAc, LDN), GalNAcβ1-4(Fucα1-3)GlcNAc (fucosylated LacdiNAc, LDN-F) and Galβ1-4(Fucα1-3)GlcNAc (Lewis X, LeX) units. These antennary modifications are expressed in schistosomes (at all developmental stages, albeit with varying surface expression patterns)^34^ but are rare in mammals^35^, and occur variably in other helminth species^19^.

Core β2Xyl and α3Fuc modified schistosome egg N-glycoproteins induce potent Th2-type cellular responses^36^. In plants, core β2Xyl and α3Fuc may be the most common N-glycan epitopes targeted by human IgE^37,38^. It is plausible that N-glycan core substitutions play a major role in the glycan-dependent host response to chronic schistosomiasis. For example, most N-glycans on the SEA-derived glycoprotein omega-1 carry core α3Fuc motifs in combination with terminal LeX units^39^. Omega-1 drives both immunoregulatory^40^ and Th2 responses^41^, the latter in a glycan-dependent manner^12^. Kappa-5, another major component of the Th2-polarising SEA^42^, expresses glycans modified with both core β2Xyl and core α3Fuc^43^. Whether protective immunity against *Schistosoma* infection and reinfection (long associated with host IgE responses^44,45^) can be credited to these epitopes will require further investigations in animal and human studies.

The advent of glycan microarray technology enabled serum/plasma profiling of antibodies raised to a wide repertoire of N-glycan variants during schistosome infections. This technology has been employed in a small number of human studies. Recently, in Ghana, sera from a few *S*. *haematobium* infected schoolchildren showed elevated IgE responses to core β2Xyl modified N-glycans on a synthetic glycan microarray^46^, and in sera from a small cohort of *Schistosoma mansoni* (*Sm*)-infected children and adults near Lake Albert, Uganda, IgG1-4 subclass responses to core β2Xyl and α3Fuc motifs were examined using the same array^47^. Two other human studies employing shotgun microarrays constructed of complex native schistosome N-glycans showed strong anti-glycan IgG and IgM responses against a wider range of N-glycans during schistosome infections^48,49^. A better understanding of population-level immune responses to *Schistosoma* glycans is important for research and clinical applications, and requires larger, well-defined immuno-epidemiological studies in endemic settings.

Fishing villages in the Lake Victoria islands of Koome, Uganda, have a high prevalence of *Sm*^50–53^, and have been surveyed as part of a portfolio of studies on helminth infections and allergy-related outcomes in Uganda. This setting provided a unique opportunity, within the context of a well-characterised large study^50^, to correlate epidemiological trends pertaining to *Sm* infection (and intensity) with microarray-detected plasma IgE and IgG responses to N-glycans with and without core α-1,3-fucosylation and/or β-1,2-xylosylation. Plasma from residents of nearby mainland urban communities with lower *Sm* exposure enabled us to make rural-urban comparisons of anti-glycan antibody responses.

## Methods

### Study design and population

Individuals included in the current investigation were randomly selected using a Stata program (StataCorp, College Station, USA) from participants of two cross-sectional surveys in rural and urban Uganda, who had a sufficient volume of stored plasma. The rural survey was the outcome survey (year three, September 2015–August 2016) of the Lake Victoria Island Intervention Study on Worms and Allergy-related diseases (LaVIISWA; ISRCTN47196031)^50^, a cluster-randomised trial of community-based standard versus intensive anthelminthic intervention in 26 *Sm*-endemic fishing villages of Koome islands (Lake Victoria, Uganda). The trial description^50,53^ and survey results after three years of intervention^52^ have been published: briefly, standard intervention included annual, community-based, mass drug administration of praziquantel; intensive intervention included quarterly praziquantel. The urban survey (September 2016–September 2017) was conducted in the 24 sub-wards of Entebbe municipality, an area with lower helminth exposure, located on the northern shores of Lake Victoria (approximately 35 km from Koome). It was designed to collect data from an urban setting for comparison with the *Sm*-endemic rural survey.

In both surveys, intestinal helminth infections were assessed using the Kato-Katz (KK) method^54^ on a single stool sample (prepared on two slides, read by different technicians). The remaining sample was stored at −80 °C and later investigated for *Sm*, *Necator americanus* and *Strongyloides stercoralis* infections using multiplex real-time PCR^55,56^. Mid-stream urine was also assessed for *Sm* circulating cathodic antigen (CCA) using a point-of-care test (Rapid Medical Diagnostics, Pretoria, South Africa). *Schistosoma haematobium* is not present in the surveyed areas^57^. Blood samples were processed to obtain plasma for immunological measurements, including N-glycan-specific IgE and IgG by microarray (detailed below) and *Schistosoma* egg [SEA]- and adult worm [SWA] antigen-specific IgE, IgG4 and IgG by ELISA (Supplementary Material).

The research ethics committees of the Uganda Virus Research Institute and the London School of Hygiene and Tropical Medicine, and the Uganda National Council for Science and Technology approved this work. All methods were performed in accordance with guidelines and regulations of these committees. Informed consent was obtained from all participants and/or their legal guardians and assent from children aged ≥8 years.

### Microarray detection of N-glycan-specific IgE and IgG

Immunoglobulin E and G responses to 135 chemically synthesised glycans with and without core α-1,3-fucosylation and, or, β-1,2-xylosylation (Supplementary Fig. S1) were assessed using a non-commercial microarray. Fluorescently-labeled bovine serum albumin (BSA) was included as an array printing control. Microarray construction procedures have been described in detail elsewhere^48,58^. The glycan antibody binding assay was adapted from existing procedures^17,46,49,59^, as follows: Nexterion H N-hydroxysuccinimide-coated microarray slides (Schott AG, Mainz, Germany) (pre-blocked with 50 mM ethanolamine in 50 mM sodium borate buffer pH 9.0, and stored at −20 °C) were thawed at room temperature (RT) and covered with silicone gaskets to create seven wells with printed microarrays per slide. Each microarray was incubated with 300 μl of a 1:30 plasma dilution in 1% BSA - 0.01% Tween20 for one hour at RT while shaking. After sequential washes with PBS-0.05% Tween20 and PBS, the slides were incubated for 30 minutes at RT in the dark with PromoFluor 647-labelled anti-human IgE (diluted 1/150 in PBS-0.01% Tween20) and Cy3-labelled anti-human IgG (diluted 1/1000 in PBS-0.01% Tween20), while shaking. After a final wash with PBS-0.05% Tween20, PBS and deionised water, sequentially, the slides were dried and kept in the dark until scanning. The slides were scanned for fluorescence at a 10μm resolution with a G2565BA scanner (Agilent Technologies, CA, USA) using 633 nm and 532 nm lasers for detection of reactivity to glycan-specific IgE and IgG, respectively.

### Data analysis

Using GenePix Pro 7.0 software (Molecular Devices, CA, USA), a spot-finding algorithm was used to align and re-size fluorescence spots in the microarray images, without setting a composite pixel intensity threshold. Data on median fluorescence intensity (MFI) for each spot and the local background were then exported to Microsoft Excel software, where background MFI subtraction was done for each glycan structure, averaged over four spots. Further processing of IgG and IgE MFIs in Excel was done as described by Oyelaran *et al*.^60^ and Amoah *et al*.^46^, respectively, to yield log_2_-transformed values.

Graphical representations of antibody responses and further data analyses were done using Stata 13.1 (College Station, Texas, USA), R (R foundation for Statistical Computing, Vienna, Austria) via the RStudio interface (version 1.1.383, RStudio, Inc. Boston, USA) and GraphPad Prism (version 6.0e, Fay Avenue, La Jolla, CA, USA). *Schistosoma mansoni* infection and the rural-urban environment were the main exposures of interest: we compared anti-glycan antibody responses between *Sm* infected and uninfected participants separately in the rural and urban survey, and thereafter between rural and urban participants. Initial analyses considered each anti-glycan antibody response independently, while further analyses combined antibody responses to reduce the dimensionality of the outcome data, as detailed below.

Rural-urban differences in *Sm* prevalence and *Schistosoma*-specific antibodies were assessed using survey design-based logistic and linear regression, respectively. Most log_2_-transformed anti-glycan IgE responses maintained a skewed distribution. Therefore, Mann-Whitney tests were used to assess differences in individual glycan structure-specific antibody responses between *Sm* infected and uninfected participants and between rural and urban participants. Most log_2_-transformed anti-glycan IgG responses were normally distributed and were assessed using unpaired t tests. The Kruskal-Wallis (IgE responses) and one-way ANOVA test (IgG responses) were also conducted to assess differences along the infection intensity gradient. Since many of the anti-glycan antibody responses were correlated, the above tests were conducted within a Monte Carlo simulation approach based on 1000 permutations, to generate empirical p-values corrected for multiple testing.

Given the large number of outcomes, two data reduction techniques were used to investigate associations between exposures and outcomes. First, principal component analysis (PCA) was run in Stata to transform groups of correlated anti-glycan responses into fewer, uncorrelated artificial variables (principal components, PCs), which were then compared by 1) survey setting, and 2) *Sm* infection and intensity status using survey design-based linear regression. Second, unsupervised hierarchical clustering analysis (HCA, complete linkage using Euclidean distance) was conducted in R to further identify homogeneous sets of N-glycan-specific responses. The resultant IgE and IgG clusters were then assessed for associations with survey setting and *Sm* infection using the global test^61–63^ executed in R with the Globaltest package (version 5.33.0).

## Results

Characteristics of the rural and urban survey participants included in this analysis are presented in Table 1. Rural participants were, on average, older [median age (IQR) 22 (5, 37)] than urban participants [median age (IQR) 11 (5, 18)] (p < 0.001). A significantly higher percentage of rural, compared to urban participants, were infected with *Sm* (KK, p = 0.002; PCR, p < 0.001; CCA, p = 0.015). Furthermore, median levels of total IgE (p < 0.001) and SEA- and SWA-specific IgE (p < 0.001), IgG4 (p = 0.001) and IgG (p = 0.002 and p < 0.001, respectively) were higher among rural compared to urban participants.

**Table 1:** Study participants: *Schistosoma mansoni* infection and *Schistosoma*-specific antibodies.

Characteristic	Rural (n = 209)	Urban (n = 62)	p value
Age in years, median (IQR)	**22 (5**, **37)**	11 (5, 18)	<0.001*
Male sex, n/N (%)^§^	97/209 (44.3)	18/62 (29.0)	0.163^¶^
**Helminth infections**, **n/N (%)**^**§**^			
*S. mansoni* (single KK)	**54/197 (34**.**5)**	4/48 (8.3)	0.002^¶^
*S*. *mansoni* intensity (KK)			0.002^¶^
Uninfected (0 eggs/g)	143/197 (65.5)	**44/48 (91**.**7)**	
Light (0–99 eggs/g)	**29/197 (17**.**2)**	3/48 (6.3)	
Moderate (100–399 eggs/g)	**14/197 (10**.**2)**	1/48 (2.1)	
Heavy (≥400 egg/g)	**11/197 (7**.**0)**	0/48 (0.0)	
*S*. *mansoni* (PCR)	**77/196 (44**.**9)**	6/48 (12.5)	<0.001^¶^
*S*. *mansoni* (urine CCA)	**118/199 (66**.**0)**	21/58 (36.2)	0.015^¶^
Any nematode infection^#^	**43/196 (18**.**7)**	1/48 (2.1)	0.001^¶^
Total IgE (kU/L), median (IQR)	**548**.**4 (404**.**4**, **666**.**9)**	103.3 (63.8, 146.5)	<0.001*
***Schistosoma*** **egg and worm*-*specific antibody levels**, **(μg/ml)**, **median (IQR)**			
SEA-specific IgE	**4**.**2 (2**.**6**, **6**.**6)**	2.2 (1.4, 3.6)	<0.001*
SWA-specific IgE	**3**.**9 (2**.**4**, **5**.**9)**	2.1 (1.3, 3.1)	<0.001*
SEA-specific IgG4	**161**.**0 (45**.**9**, **663**.**8)**	8.8 (0.0, 48.4)	0.001*
SWA-specific IgG4	**71**.**6 (39**.**5**, **188**.**1)**	32.1 (7.8, 57.9)	0.001*
SEA-specific IgG	**1687**.**3 (848**.**1**, **2727**.**7)**	730.7 (527.4, 1413.4)	0.002*
SWA-specific IgG	**1432**.**4 (845**.**8**, **1941**.**6)**	804.5 (572.7, 1311.3)	<0.001*

^§^Percentages adjusted for survey design.

^¶^P values obtained from survey design-based logistic regression.

*P values obtained from survey design-based linear regression.

^#^Infection with any of *Strongyloides stercoralis*, *Necator americanus* (assessed by PCR), *Trichuris trichiura*, *Ascaris lumbricoides* (assessed by KK) and *Mansonella perstans* (assessed by modified Knott’s method).

Percentages/medians that are significantly higher in one setting compared to the other (p ≤ 0.05) are highlighted in bold.

KK: Kato-Katz; PCR: Polymerase Chain Reaction; CCA: Circulating Cathodic Antigen; IQR: Interquartile range; SEA: *Schistosoma* egg antigen; SWA: *Schistosoma* adult worm antigen.

We recently reported that community-based intensive versus standard anthelminthic intervention in the rural survey reduced *Sm* infection intensity but had no effect on the overall *Sm* prevalence (measured using the urine CCA test)^52^. The current analysis found no evidence of an effect of intensive versus standard treatment on total IgE, SEA- or SWA-specific antibodies, or on antibody reactivity to any of the N-glycans on the microarray. Therefore, data from the rural survey were not stratified by trial treatment arm in the further analyses presented herein.

### Associations between *S*. *mansoni* infection and IgE and IgG responses to individual core β-1,2-xylosylated and core α-1,3-fucosylated N-glycans

**Figure 2:**
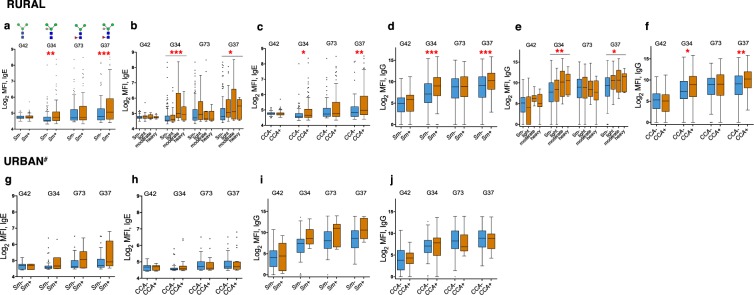
Associations between *S. mansoni* infection and IgE and IgG reactivity to N-glycans carrying non-mammalian core modifications. Plasma from *S. mansoni* infected and uninfected rural and urban individuals were assessed for IgE and IgG reactivity to N-glycan structural variants with and without α-1,3-fucosylation and β-1,2-xylosylation, on a microarray platform. Box-and-whisker plots show background-subtracted and log2-transformed median fluorescence intensities (MFI) representing IgE (**a–c**,**g**,**h**) and IgG (**d–f**,**i**,**j**) reactivity to the Man_3_GlcNAc_2_ core structure (G42) and to α3Fuc- and/or β2Xyl-carrying Man_3_GlcNAc_2_ core structures (G34, G73 and G37). The plots show a horizontal line denoting the median, a box indicating the interquartile range (IQR), and whiskers drawn using the Tukey method (1.5 times IQR). Outliers (greater than 1.5 times IQR away from the median) are plotted as individual points. Mann-Whitney (IgE responses) and unpaired t test (IgG responses) were conducted within the framework of a Monte Carlo simulation algorithm based on 1000 permutations (in order to adjust for multiple testing), to assess differences between infected and uninfected individuals. The Kruskal-Wallis (IgE responses) and one-way ANOVA test (IgG responses) were also conducted using the permutation approach to assess differences along the infection intensity gradient (**b** and **e**) in the rural survey. ^#^Infection prevalence and intensity was relatively low in the urban survey so analysis by *Sm* intensity is not shown for the urban setting. *p < 0.05; **p < 0.01; ***p < 0.001. Sm: *S. mansoni* infection determined by detection of eggs in a single stool sample by Kato-Katz and/or PCR (rural infected n = 84, uninfected n = 113; urban infected n = 6, uninfected n = 42); CCA: *S. mansoni* infection determined by a positive urine circulating cathodic antigen (CCA) result (rural infected n = 118, uninfected n = 81; urban infected n = 21, uninfected n = 37).

In the rural survey, IgE and IgG responses to the β2Xyl modified Man_3_GlcNAc_2_ core (G34) were significantly higher among *Sm* infected (KK and/or PCR, and CCA positive), compared to uninfected individuals, and were positively associated with *Sm* infection intensity (KK) [Fig. 2a–f] and SWA- and SEA-specific IgE and IgG (Supplementary Table S1**)**. Observations were similar for the N-glycan core carrying both β2Xyl and α3Fuc (G37). However, IgE and IgG responses to the N-glycan core carrying α3Fuc only (G73) were similar between *Sm* infected and uninfected rural individuals (Fig. 2a–f), but positively associated with SWA- and SEA-specific IgE and IgG (Table S1).

In the urban survey, IgE and IgG reactivity to core β2Xyl and/or core α3Fuc modified glycans was also higher in *Sm* infected (KK and/or PCR) compared to uninfected individuals, although differences were not statistically significant (Fig. 2g–j). However, IgE reactivity to core α3Fuc and core α3Fuc + core β2Xyl modified glycans was significantly positively associated with SEA- and SWA-specific IgE (Table S1).

**Figure 3:**
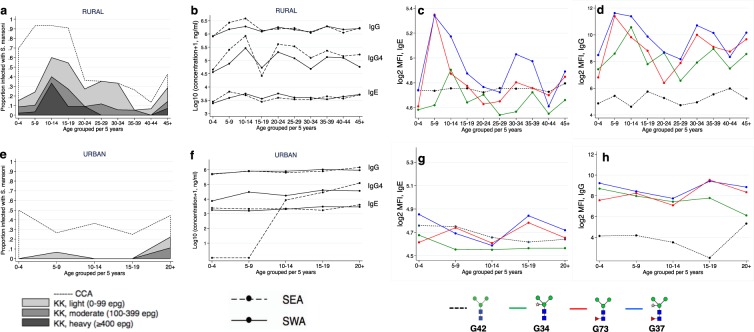
Age-stratified prevalence of *S. mansoni* infection and median IgE and IgG reactivity to SWA, SEA and α-1,3-fucosylated and β-1,2-xylosylated N-glycans. (**a**,**e**) Rural and urban prevalence and intensity of *S. mansoni* infection, by age group. (**b**,**f**) Median IgG, IgG4 and IgE reactivity to SWA and SEA, by age group, among rural and urban participants, respectively. (**c**,**g**) Median IgE reactivity to the Man_3_GlcNAc_2_ core and to α3Fuc- and/or β2Xyl modified Man_3_GlcNAc_2_ core structures, by age group, among rural and urban participants, respectively. Plotted results are from all participants, irrespective of *Sm* infection status. (**d**,**h**) Median IgG reactivity to the Man_3_GlcNAc_2_ core and to α3Fuc- and/or β2Xyl-modified Man_3_GlcNAc_2_ core structures, by age group, among rural and urban participants, respectively. Plotted results are from all participants, irrespective of *Sm* infection status. CCA: *S. mansoni* infection determined by a positive urine circulating cathodic antigen (CCA) result; KK: *S. mansoni* infection determined by detection of eggs in a single stool sample by Kato-Katz (KK); epg: Eggs per gram of stool; SWA: *Schistosoma* adult worm antigen; SEA: *Schistosoma* egg antigen.

In the rural survey, *Sm* infection prevalence (and intensity) and median levels of *Schistosoma*-specific antibodies (except SEA-IgE) and β-1,2-xylosylated glycan (G34)-specific IgE and IgG were highest among 10-14-year old individuals (Fig. 3). However, IgE and IgG reactivity to glycans carrying either core α3Fuc (G73) or both core β2Xyl and core α3Fuc (G37) peaked earlier (in the 5-9-year age group), akin to SEA-specific IgE. Age-stratified antibody reactivity patterns were less clear in the urban survey.

Immunoglobulin E and G responses to other N-glycan structural variants with core β2Xyl or both core β2Xyl and core α3Fuc^48^ were also higher in *Sm* infected versus uninfected individuals (Fig. S2). IgE and IgG reactivity to non-xylosylated and non-fucosylated glycans was not associated with *Sm* infection (data not shown), except for those glycans with antennae constructed of LDN-F (G90) and LeX (G89) units (Fig. S3).

Infection with other helminths, malaria or HIV was not associated with IgE or IgG reactivity to any glycans on the microarray (data not shown).

### Principal component analysis of anti-glycan antibody responses

Antibody responses to individual core modified N-glycans were strongly correlated. Principal component analysis (PCA) was conducted to summarise these responses, and to evaluate to what extent the resultant principal components (PCs) were associated with *Sm* infection.

**Figure 4:**
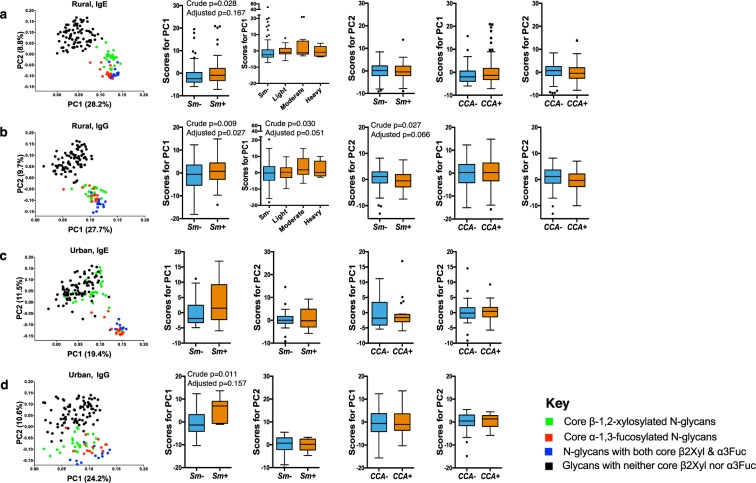
Principal component analysis of anti-glycan antibody responses. Scatterplots of first (PC1) and second factor (PC2) loadings derived from principal component analysis of IgE and IgG responses to 135 synthetic N-glycans. Box-and-whisker plots show comparison of PC1 and PC2 scores between *S. mansoni* infected and uninfected individuals. The plots show a horizontal line denoting the median, a box indicating the interquartile range (IQR), and whiskers drawn using the Tukey method (1.5 times IQR). Outliers (greater than 1.5 times IQR away from the median) are plotted as individual points. Panels a and b show IgE and IgG profiles, respectively, among rural participants. Panels c and d show IgE and IgG profiles, respectively, among urban participants. Associations between factor loading scores and *S. mansoni* infection and intensity were assessed by linear regression analysis in Stata 13.1. Crude and age- and sex-adjusted p values are shown for significant associations. All analyses were adjusted for survey design using the ‘svy’ command in Stata. PC1: Principal Component 1; PC2: Principal Component 2; Sm: *S. mansoni* infection determined by detection of eggs in a single stool sample by Kato-Katz and/or PCR (rural infected n = 84, uninfected n = 113; urban infected n = 6, uninfected n = 42); CCA: *S. mansoni* infection determined by a positive urine circulating cathodic antigen (CCA) result (rural infected n = 118, uninfected n = 81; urban infected n = 21, uninfected n = 37).

Scatterplots of PC1 and PC2 loadings are shown in Fig. 4. In the rural survey, the first two IgE and IgG PCs each accounted for 37% of the total variance in the data (IgE: PC1 28.2%, PC2 8.8%; IgG: PC1 27.7%, PC2 9.7%). Principal component 1 was characterized by responses to core β2Xyl and/or α3Fuc modified glycans while PC2 was characterized by responses to non-xylosylated and non-fucosylated glycans (Fig. 4, panel a and b). Scores for IgE PC1, but not PC2, were higher among *Sm* infected (KK or PCR) compared to uninfected individuals (crude p = 0.028, age- and sex-adjusted p = 0.167). Similarly, IgG PC1 scores were higher among *Sm* infected (KK or PCR) compared to uninfected individuals (crude p = 0.009, adjusted p = 0.027). There were no differences in PC scores between CCA+ and CCA− individuals.

In the urban survey, the first two IgE and IgG PCs accounted for 31% and 35% of the total variance, respectively (IgE: PC1 19.4%, PC2 11.5%; IgG: PC1 24.2%, PC2 10.6%). Interestingly, most IgE responses to glycans carrying core β2Xyl without α3Fuc clustered with non-xylosylated and non-fucosylated glycans in PC2 while responses to glycans carrying both core β2Xyl and α3Fuc and those carrying core α3Fuc without β2Xyl clustered together in PC1 (Fig. 4, panel c). Akin to the rural survey, scores for IgE and IgG PC1 were higher among *Sm* infected compared to uninfected urban individuals.

Scores for PC1 were positively associated with SWA- and SEA-specific IgE and IgG in both surveys, while PC2 scores were inversely associated with the same *Schistosoma*-specific antibodies (Table S1).

In addition to PCA, we conducted HCA to further identify groups of anti-glycan IgE and IgG responses that might be jointly elicited in *Sm* infected versus uninfected individuals. Figure S4 shows clusters of IgE and IgG responses in the rural and urban survey, and the dominant core substitutions on the glycans in these clusters. Generally, antibody clusters comprising core β2Xyl modified glycans were positively associated with *Sm* infection and intensity in both surveys (Table S2).

### Rural-urban comparisons of anti-glycan antibody responses

**Figure 5:**
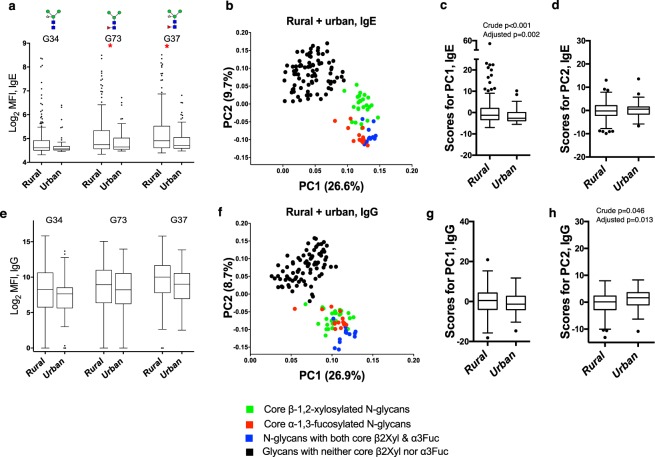
Rural-urban comparisons of anti-glycan antibody responses. (**a**,**e**) Box-and-whisker plots showing background-subtracted and log2-transformed median fluorescence intensities (MFI) representing IgE and IgG reactivity to individual α3Fuc- and/or β2Xyl-carrying Man_3_GlcNAc_2_ core structures in rural versus urban participants. The plots show a horizontal line denoting the median, a box indicating the interquartile range (IQR), and whiskers drawn using the Tukey method (1.5 times IQR). Outliers (greater than 1.5 times IQR away from the median) are plotted as individual points. Mann-Whitney (IgE responses) and unpaired t test (IgG responses) were conducted within the framework of a Monte Carlo simulation algorithm based on 1000 permutations, to assess differences between rural and urban individuals. (**b**,**f**) First and second principal component loadings of N-glycan-specific IgE and IgG responses among all participants, irrespective of survey setting. **(c**,**d**,**g**,**h**) Box-and-whisker plots showing comparison of PC1 and PC2 scores between rural and urban individuals. Associations between PC scores and survey setting were assessed by linear regression in Stata 13.1. Crude and age- and sex-adjusted p values are shown for significant associations. All analyses were adjusted for survey design using the ‘svy’ command in Stata. *p < 0.05; **p < 0.01; ***p < 0.001. PC1: Principal Component 1; PC2: Principal Component 2.

Immunoglobulin E responses to individual core β2Xyl and/or α3Fuc modified glycans were higher among rural compared to urban participants, as exemplified in Fig. 5a. Principal component analysis of data combined from both surveys yielded distinct groups of anti-glycan responses (Fig. 5b,f): PC1 was characterized by responses to core β2Xyl and/or α3Fuc modified glycans while PC2 was characterized by responses to non-xylosylated and non-fucosylated glycans. Scores for IgE PC1 (Fig. 5c), but not PC2 (Fig. 5d), were higher among rural compared to urban individuals (p = 0.002). Differences in IgG PC1 scores were not statistically significant. However, IgG PC2 scores were lower among rural compared to urban individuals (p = 0.013).

Further assessment by HCA showed that clusters that comprised IgE responses to core β2Xyl and/or α3Fuc modified glycans (IgE-C1, IgE-C2 and IgE-C4; Fig. S5) were positively associated with the rural setting (Table S4), while IgE-C3 (characterised by very low responses, raised against non-xylosylated and non-fucosylated glycans) was positively associated with the urban setting. Immunoglobulin G response clusters were generally similar between rural and urban settings, except for IgG-C7 which comprised responses to non-xylosylated and non-fucosylated glycans and was positively associated with the urban setting.

## Discussion

By studying rural *Sm-*endemic Ugandan fishing communities and a proximate urban community, we have dissected antibody responses to core β2Xyl and α3Fuc modified N-glycans. Antibody responses to the core modified glycans were higher in the rural communities compared to a proximate urban community. In the urban community, IgE and IgG to both core β2Xyl and core α3Fuc were positively associated with *Sm* infection. In the rural communities, IgE and IgG to core β2Xyl were strongly positively associated with *Sm* infection while reactivity to core α3Fuc was elevated in both *Sm* infected and uninfected individuals. In the rural communities the concentration of antibodies to core α3Fuc modified N-glycans peaked ahead of the peak of *Sm* infection intensity, while the peak of antibodies to N-glycans with only core β2Xyl coincided with it.

The positive association between current *Sm* infection and IgE and IgG reactivity to N-glycans carrying only core α3Fuc in the urban, but not the rural communities, might reflect universal exposure to infection, and persistence of light infection despite treatment, in the rural setting. Core α3Fuc is abundant on N-glycans from *Sm* eggs but is not expressed by cercarial and adult worm N-glycans^19,20,24^. It is plausible that responses to core α3Fuc persist after active infection in high *Sm* exposure rural settings: in mice, eggs and hepatic granulomas persist long after clearance of worms^64^. Another explanation for elevated responses to α3Fuc in the rural communities, regardless of *Sm* infection status, is cross-reactivity. Core α-1,3-fucosylation and β-1,2-xylosylation are also present on certain plant and insect glycoproteins^29,30,65^, hence similar core α3Fuc responses in both *Sm* infected and uninfected individuals may also be explained by an exposure other than schistosomes, more prevalent in the rural than the urban setting, that carries core α3Fuc. The observation that antibodies to core β2Xyl were significantly higher among *Sm* infected individuals in both urban and rural settings implies a dominant role for core β2Xyl (compared to core α3Fuc) in *Sm*-specific humoral immunity, shown here for the first time. It also appears that responses only to core β2Xyl are more responsive to change in *Sm* exposure: core β2Xyl is abundant on cercarial N-glycans despite being absent in adult worms.

The prominent contribution of core β2Xyl and α3Fuc to cross-reactivity between schistosomes and other environmental exposures such as pollen, hymenoptera venom and vegetable foods^22,37,38^ is a caveat against the use of core modified glycans in schistosome diagnostic tests. Cross-reactivity with other helminth infections might also occur, but only a few other helminth species^25–28^, none of which are prevalent in humans in our survey settings, have so far been demonstrated to express glycans with core β2Xyl and α3Fuc motifs. More extensive glycomic studies of other helminths in our survey settings (*S*. *stercoralis*, hookworm, *T*. *trichiura*, *A*. *lumbricoides*, *M*. *perstans*) are warranted. However, we did not find any significant associations between these infections and IgE or IgG reactivity to core modified glycans.

Our observations that IgE and IgG reactivity to N-glycans modified with antennae carrying LDNF and LeX units were associated with *Sm* infection in both surveys are consistent with previous studies in animal models and in humans^49,66^. No associations were observed with responses to glycans carrying unsubstituted LDN units.

Principal component analysis indicated strong correlations between antibody responses to β-1,2-xylosylated glycans and responses to α-1,3-fucosylated glycans in both surveys. Core β2Xyl and α3Fuc epitopes can be found on similar *Sm* antigens, where they may be expressed on the same glycoproteins and glycans (such as those expressed by SEA)^19^, inducing analogous immune responses^36^. Non-xylosylated and non-fucosylated glycans with antennae constructed of LDN, LDN-F or LeX units may be expressed on the same *Sm* antigens as glycans with core β2Xyl and α3Fuc motifs. Furthermore, these terminal antennary substitutions can occur on the same glycans as core β2Xyl and α3Fuc^19,39,43^. However, PCA showed that responses to non-core-substituted glycans with LDN, LDN-F or LeX units did not cluster with core β2Xyl/α3Fuc substituted glycans. Temporal changes in expression of glycans on *Sm* antigens have been reported^20,34^; it is possible that these two groups of glycans are expressed at varying magnitudes during *Sm* antigen maturation. Positive associations between *Sm* infection and the first principal component (representing responses to core β2Xyl and α3Fuc) reflect the important role of these core substitutions in the glycan-dependent host response to *Sm*. To further evaluate their contribution to the host immune response to *Sm*, it will be important to compare their antibody reactivity with that of other highly antigenic terminal motifs absent from glycans on the array used in this study, such as multi-fucosylated LDN motifs^59^. It is important to note that while IgG is abundantly detected to many schistosome glycans, and is triggered by *Sm* infection, only to core β2Xyl and α3Fuc modified glycans is IgE abundantly detected^46,47,67^.

Notably in the urban survey, PCA showed that IgE responses to core β2Xyl modified glycans clustered with responses to non-xylosylated and non-fucosylated glycans (Fig. 4c), and IgG responses to core β2Xyl and α3Fuc separated out less distinctly (Fig. 4d) than in the rural survey. The observed β2Xyl clustering patterns may be attributed to the greater intensity of repeated exposure to schistosome cercariae (core β2Xyl) and sustained egg deposition (core β2Xyl and α3Fuc) among rural compared to urban participants. In other words, rural-urban differences in antibody responses to core modified glycans may be indicative of differences in the intensity of *Sm* infection and/or degree of exposure between the two settings. However, this study did not have sufficient power to assess statistical interactions between the rural and the urban setting. Rural-urban differences in antibody responses to core modified glycans may also be explained by exposures other than schistosomes (mentioned above), perhaps more prevalent in the rural than the urban setting; however, this is unlikely as we observed strong associations between *Sm* infection and reactivity to core modified N-glycans, particularly to those carrying core β2Xyl. It is also noteworthy that urban survey participants were significantly younger than rural participants; however, this disparity did not seem to influence the observed rural-urban differences in anti-glycan responses, as observed from test statistics before and after adjusting for age.

One of the key challenges in schistosomiasis vaccine development is the risk of allergic (IgE) sensitisation to candidate vaccine antigens^68^. Glycans are attractive vaccine candidates because they are generally considered to be benign as allergenic determinants^69,70^. There are a few known exceptions, such as the galactose-α-1,3-galactose (α-1,3-gal) epitope (found in non-primate mammalian proteins, and shown to elicit severe allergy)^71^, so assessment for any associations between IgE to antigenic *Sm* glycans and allergy-related phenomena are important. The case for consideration of core modified *Sm* glycans as *Schistosoma* vaccine candidates will also need definite proof for an association between reactivity to core modified glycans and protection from *Sm* infection/re-infection. Our data suggests that a protective role, if any, is more plausible for core β2Xyl than core α3Fuc: in the rural survey, antibody responses to core α3Fuc (G73 and G37, Fig. 3c,d) peaked in childhood, prior to the *Sm* infection peak in early adolescence, while responses to core β2Xyl (G34) coincided with the *Sm* infection peak (preceding the more ‘protected’ period in adulthood). However, concrete evidence is required from further population and mechanistic studies exploring the role of *Sm* N-glycans in protective immunity. For example, it may be important to assess antibodies to these core modifications (and other antigenic terminal motifs) in re-infection study cohorts evaluating the immunological characteristics of individuals who are *Sm-*resistant following anthelminthic treatment.

In conclusion, we provide an immuno-epidemiological description of IgE and IgG responses to N-glycans in rural and urban Uganda, highlighting the significance of core β2Xyl and core α3Fuc to the glycan-dependent host immune response during chronic schistosomiasis. Moreover, our data imply that IgE and IgG responses to core β2Xyl and α3Fuc modified N-glycans have distinctive relationships with *Sm* infection and intensity, which may reflect their different contributions towards protective immunity against *Sm* that need to be further explored using mechanistic animal and human studies.

## Supplementary information

Supplementary information

## Data Availability

The datasets generated during and/or analysed during the current study are available from the corresponding author on reasonable request.
