# Supplementary material for: Microarray assessment of N-glycan-specific IgE and IgG profiles associated with Schistosoma mansoni infection in rural and urban Uganda
Source: Sci Rep. 2019 Mar 5;9:3522. doi: 10.1038/s41598-019-40009-7 (PMC6401159; doi:10.1038/s41598-019-40009-7)
Supplement: Supplementary file 1 — Supplementary information [file 41598_2019_40009_MOESM1_ESM.docx]

**Microarray assessment of N-glycan-specific IgE and IgG profiles associated with *Schistosoma mansoni* infection in rural and urban Uganda**

Gyaviira Nkurunungi^1,2§^, Angela van Diepen^3^, Jacent Nassuuna^1^, Richard E Sanya^1,4^, Margaret Nampijja^1^, Irene Nambuya^1^, Joyce Kabagenyi^1^, Sonia Serna^5^, Niels-Christian Reichardt^5,6^, Ronald van Ree^7^, Emily L Webb^8^, Alison M Elliott^1,2^*, Maria Yazdanbakhsh^3^*, Cornelis H Hokke^3§^*

^1^Immunomodulation and Vaccines Programme, Medical Research Council / Uganda Virus Research Institute and London School of Hygiene and Tropical Medicine (MRC/UVRI and LSHTM) Uganda Research Unit, Entebbe, Uganda

^2^Department of Clinical Research, London School of Hygiene and Tropical Medicine, London, United Kingdom

^3^Department of Parasitology, Leiden University Medical Center, Leiden, The Netherlands

^4^College of Health Sciences, Makerere University, Kampala, Uganda

^5^Glycotechnology Laboratory, Centro de Investigación Cooperativa en Biomateriales (CIC biomaGUNE), San Sebastián, Spain

^6^Centro de Investigación Biomédica en Red en Bioingeniería, Biomateriales y Nanomedicina (CIBER-BBN), San Sebastián, Spain

^7^Amsterdam University Medical Centers, Departments of Experimental Immunology and of Otorhinolaryngology, Amsterdam, The Netherlands

^8^MRC Tropical Epidemiology Group, Department of Infectious Disease Epidemiology, London School of Hygiene and Tropical Medicine, London, United Kingdom

^§^Correspondence and requests for materials should be addressed to G.N. (email: [gyaviira.nkurunungi@mrcuganda.org](mailto:gyaviira.nkurunungi@mrcuganda.org)) and C.H.H. (email: [C.H.Hokke@lumc.nl](mailto:C.H.Hokke@lumc.nl))

*****These authors contributed equally to this work.

**SUPPLEMENTARY INFORMATION**

1. **Experimental methods**

## S. mansoni adult worm (SWA)- and egg (SEA)-specific IgE and IgG4 ELISA

All but the first 2 columns of 4HBX Immulon (Thermo Scientific, NY, USA) 96-well plates were coated with 50μl of SWA [8 μg/ml] or SEA [2.4 μg/ml] (purchased from Professor Michael J Doenhoff, University of Nottingham) in bicarbonate (Na_2_CO_3_ + NaHCO_3_) buffer (0.1M, pH 9.6). Two-fold dilutions of human IgE (Calbiochem, Beeston, UK) or IgG4 (Sigma-Aldrich) standard, diluted in bicarbonate buffer, were added to the first 2 columns of each plate to form standard curves. The plates were then incubated overnight at 4ºC. Plates were washed with phosphate-buffered saline (PBS 1X)-tween 20 solution, blocked with 150μl of 1% skimmed milk diluted in PBS-Tween 20 at room temperature (RT), and incubated overnight at 4ºC with 50μl of plasma samples diluted 1/20 (IgE assay) or 1/200 (IgG4 assay) with 0.1% skimmed milk in PBS-Tween 20 (assay buffer). Plates were washed and antibody binding detected by incubating the plates overnight at 4ºC with 0.5μg/ml of biotinylated monoclonal mouse anti-human IgE or IgG4 (BD Pharmingen™). This was followed by a 1-hour incubation with a streptavidin-Horseradish Peroxidase (strep-HRP) conjugate (Mast Group Ltd, Bootle, UK), diluted 1/4000 with assay buffer, at RT. Plates were developed by addition of 100μl of o-phenylenediamine (Sigma-Aldrich) and reactions stopped after 30 minutes with 25μl of 2M Sulphuric acid. Optical density values were measured at 490nm (reference wavelength 630nm) on a 96-well plate ELISA reader. IgE or IgG4 concentrations (ng/ml) were interpolated from standard curves using a five-parameter curve fit using Gen5 data collection and analysis software (BioTek Instruments Inc, Vermont, Winooski, USA).

## S. mansoni adult worm (SWA)- and egg (SEA)-specific IgG ELISA

All but the first 2 columns of 4HX Immulon (VWR, UK, Cat No 735-0465) 96-well plates were coated with 50μl of SWA [8 μg/ml] or SEA [2.4 μg/ml] (purchased from Professor Michael J Doenhoff, University of Nottingham) in bicarbonate (Na_2_CO_3_ + NaHCO_3_) buffer (0.1M, pH 9.6). Two-fold dilutions of human IgG (Sigma-Aldrich), diluted in bicarbonate buffer, were added to the first 2 columns of each plate to form standard curves. The plates were then incubated overnight at 4ºC. Plates were washed with phosphate-buffered saline (PBS)-Tween 20 solution, blocked with 150μl of 1% skimmed milk diluted in PBS-Tween 20 at room temperature (RT), and incubated overnight at 4ºC with 50μl of plasma samples diluted 1/3000 with 0.1% skimmed milk in PBS-Tween 20 (assay buffer). Plates were washed and antibody binding detected by incubating the plates for 1 hour at RT with 0.5μg/ml of polyclonal rabbit anti- human IgG/HRP (Dako, Denmark). Plates were developed by addition of 100μl of o-phenylenediamine (Sigma-Aldrich) and reactions stopped after 30 minutes with 25μl of 2M Sulphuric acid. Optical density values were measured at 490nm (reference wavelength 630nm) on a 96-well plate ELISA reader. IgG concentrations (ng/ml) were interpolated from standard curves using a five-parameter curve fit using Gen5 data collection and analysis software (BioTek Instruments Inc, Vermont, Winooski, USA).

1. **Supplementary figures and tables**

**Figure S1.** Synthetic N-glycan structural variants on the microarray

| 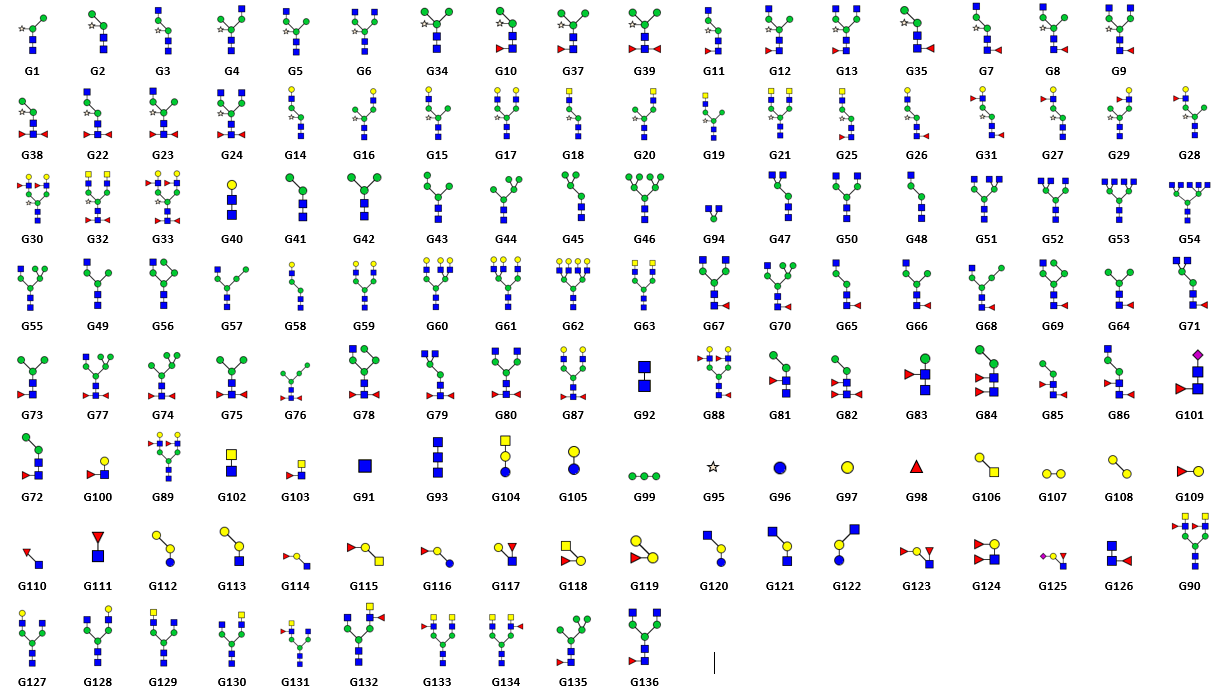 |
| --- |
| 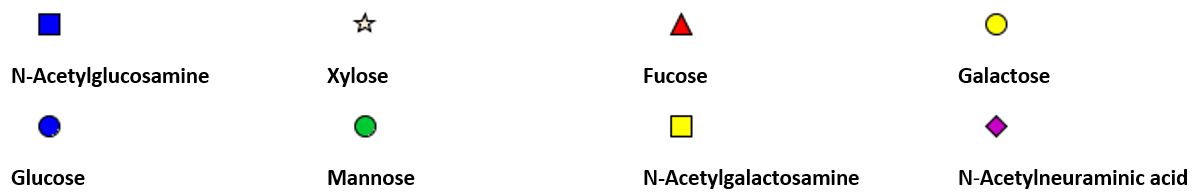 |

**Table S1.** Associations between anti-glycan and *Schistosoma* adult worm- and egg*-*specific antibody responses

|  |  | *Associations between anti-glycan IgE & anti-Sm IgE* | | | |  | *Associations between anti-glycan IgG & anti-Sm IgG* | | | |
| --- | --- | --- | --- | --- | --- | --- | --- | --- | --- | --- |
|  | **Glycan** | **SEA-specific IgE** | | **SWA-specific IgE** | |  | **SEA-specific IgG** | | **SWA-specific IgG** | |
|  |  | *β (95% CI)* | *p* | *β (95% CI)* | *p* |  | *β (95% CI)* | *p* | *β (95% CI)* | *p* |
| RURAL | 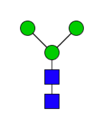  G42 | -0.02  (-0.07, 0.03) | 0.364 | -0.00  (-0.04, 0.04) | 0.927 |  | 1.06  (-0.11, 2.24) | 0.075 | **3.06**  **(1.95, 4.16)** | **<0.001** |
| URBAN |  | 0.1  (0.0, 0.1) | 0.050 | 0.1  (-0.2, 0.3) | 0.660 |  | 0.48  (-2.05, 3.01) | 0.678 | -0.68  (-4.36, 3.01) | 0.688 |
| RURAL | 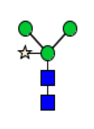  G34 | **0.76**  **(0.36, 1.16)** | **0.001** | **0.86**  **(0.60, 1.11)** | **<0.001** |  | **2.67**  **(0.67, 4.68)** | **0.011** | **5.66**  **(3.26, 8.07)** | **<0.001** |
| URBAN |  | 0.1  (-0.1, 0.3) | 0.165 | 0.78  (-0.02, 1.58) | 0.056 |  | 1.43  (-1.06, 3.92) | 0.225 | 1.49  (-2.07, 5.05) | 0.369 |
| RURAL | 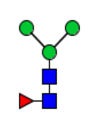  G73 | **0.81**  **(0.36, 1.26)** | **0.001** | 0.52  (-0.29, 1.33) | 0.205 |  | **1.61**  **(0.05, 3.16)** | **0.044** | **2.71**  **(0.66, 4.75)** | **0.012** |
| URBAN |  | **0.25**  **(0.02, 0.47)** | **0.036** | **1.01**  **(0.31, 1.69)** | **0.009** |  | 1.62  (-1.29, 4.54) | 0.239 | 1.21  (-1.75, 4.18) | 0.379 |
| RURAL | 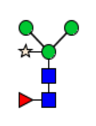  G37 | **1.13**  **(0.52, 1.74)** | **0.001** | **1.07**  **(0.39, 1.74)** | **0.003** |  | **2.26**  **(0.44, 4.08)** | **0.017** | **4.55**  **(3.46, 5.65)** | **<0.001** |
| URBAN |  | **0.29**  **(0.04, 0.56)** | **0.029** | **1.61**  **(0.88, 2.33)** | **0.001** |  | 1.79  (-1.52, 5.11) | 0.251 | 1.95  (-1.53, 5.45) | 0.236 |
| RURAL | 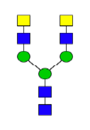  G63 | -0.01  (-0.11, 0.08) | 0.762 | 0.01  (-0.09, 0.11) | 0.865 |  | 0.32  (-0.11, 0.75) | 0.137 | **0.99**  **(0.19, 1.78)** | **0.016** |
| URBAN |  | **0.05**  **(0.01, 0.11)** | **0.025** | 0.15  (-0.16, 0.46) | 0.316 |  | 0.36  (-1.30, 2.03) | 0.632 | 0.55  (-1.83, 2.95) | 0.609 |
| RURAL | 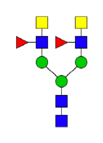  G90 | **0.04**  **(0.02, 0.05)** | **<0.001** | **0.05**  **(0.03, 0.08)** | **<0.001** |  | **1.39**  **(0.19, 2.59)** | **0.025** | **3.41**  **(2.09, 4.73)** | **<0.001** |
| URBAN |  | -0.00  (-0.02, 0.01) | 0.437 | -0.00  (-0.04, 0.04) | 0.890 |  | **2.68**  **(0.97, 4.37)** | **0.006** | **3.24**  **(1.00, 5.47)** | **0.010** |
| RURAL | 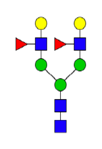  G89 | 0.04  (-0.03, 0.04) | 0.813 | 0.01  (-0.04, 0.49) | 0.815 |  | 1.24  (-0.04, 2.51) | 0.057 | **3.40**  **(1.11, 5.68)** | **0.005** |
| URBAN |  | 0.00  (-0.01, 0.01) | 0.980 | -0.04  (-0.09, 0.01) | 0.118 |  | **2.46**  **(0.33, 4.59)** | **0.028** | **3.51**  **(1.24, 5.77)** | **0.007** |
| RURAL | PC1 | **6.51**  **(2.69, 10.34)** | **0.002** | **5.85**  **(1.80, 9.91)** | **0.006** |  | **4.27**  **(0.22, 8.33)** | **0.040** | **9.96**  **(6.75, 13.18)** | **<0.001** |
| URBAN |  | 1.93  (-1.39, 5.25) | 0.222 | **9.48**  **(2.66, 16.31)** | **0.012** |  | 5.02  (-4.49, 14.5) | 0.263 | 5.88  (-3.58, 15.3) | 0.193 |
| RURAL | PC2 | **-2.74**  **(-4.64, -0.83)** | **0.007** | -1.15  (-3.25, 0.92 | 0.263 |  | **-1.82**  **(-3.41,-0.23)** | **0.027** | **-2.64**  **(-4.46, -0.81)** | **0.006** |
| URBAN |  | -1.89  (-4.33, 0.55) | 0.114 | -5.15  (-11.2, 0.86) | 0.084 |  | -0.89  (-2.94, 1.15) | 0.347 | -0.87  (-0.28, 1.15) | 0.354 |
| *Table shows regression coefficients (β) and corresponding 95% confidence intervals (95% CI) and p-values for associations between anti-glycan antibody responses and Schistosoma adult worm (SWA)- and egg (SEA)-specific antibody responses, among rural and urban participants. The Man_3_GlcNAc_2_ core structure (G42), α3Fuc- and/or β2Xyl-carrying Man_3_GlcNAc_2_ core structures (G34, G73 and G37) and N-glycan core structures carrying LDN (G63), LDNF (G90) and Lewis X (G89) antennae were chosen to represent the wide range of Schistosoma-associated N-glycans on the array. PC1 and PC2 denote first and second factor loadings derived from principal component (PC) analysis of IgE and IgG responses to the 135 synthetic N-glycans. PC1 was characterised by responses to core β2Xyl and/or core α3Fuc modified glycans while PC2 was characterised by responses to non-xylosylated and non-fucosylated glycans (Figure 4 in main paper, panels A and B).*  *All associations are adjusted for the survey design using the ‘svy’ command in Stata 13.1, and for age and sex.* | | | | | | | | | | |

**Figure S2.** Associations between *S. mansoni* infection (KK and/or PCR) and IgE and IgG reactivity to N-glycan structural variants carrying α1-3 fucose and/or β1-2 xylose

| **RURAL SURVEY** |
| --- |
| **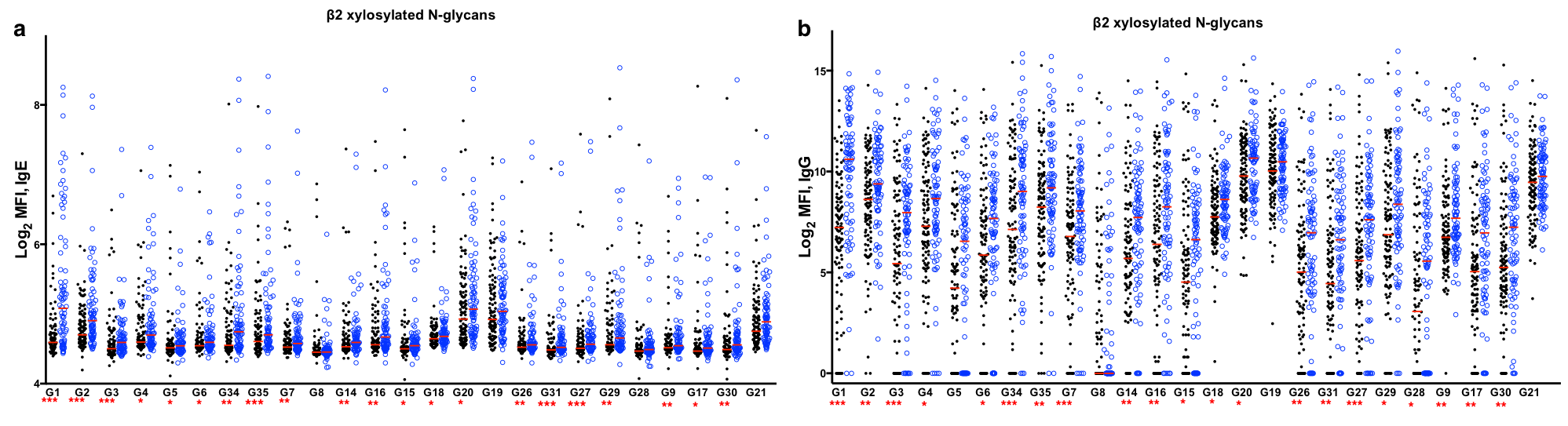 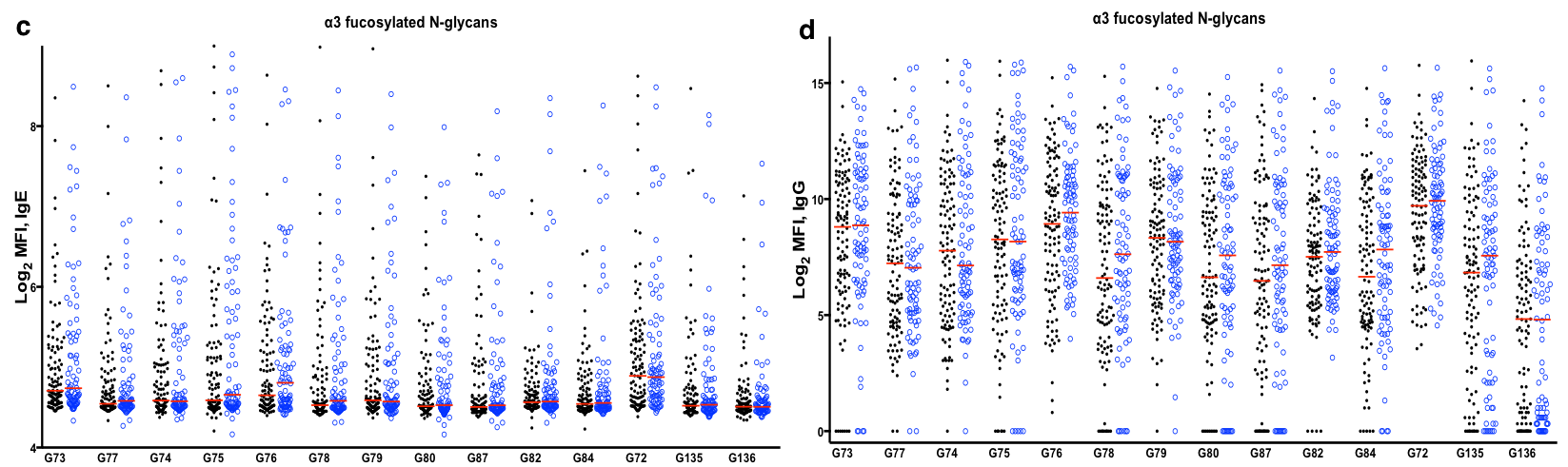**  **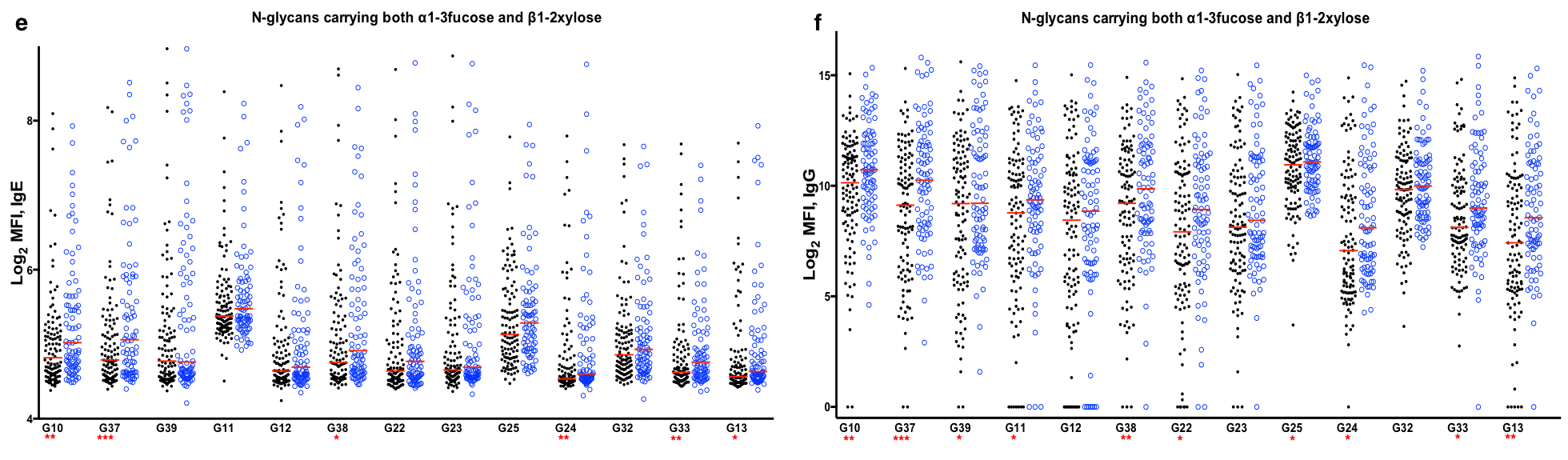**  **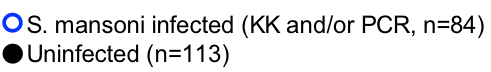** |
| **URBAN SURVEY** |
| **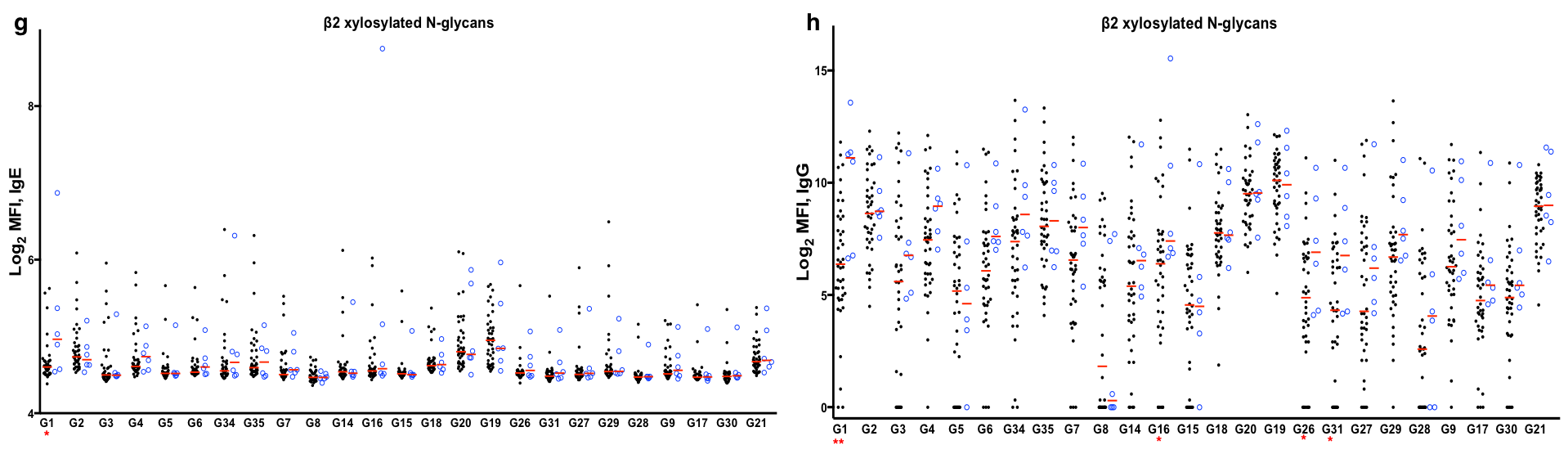**  **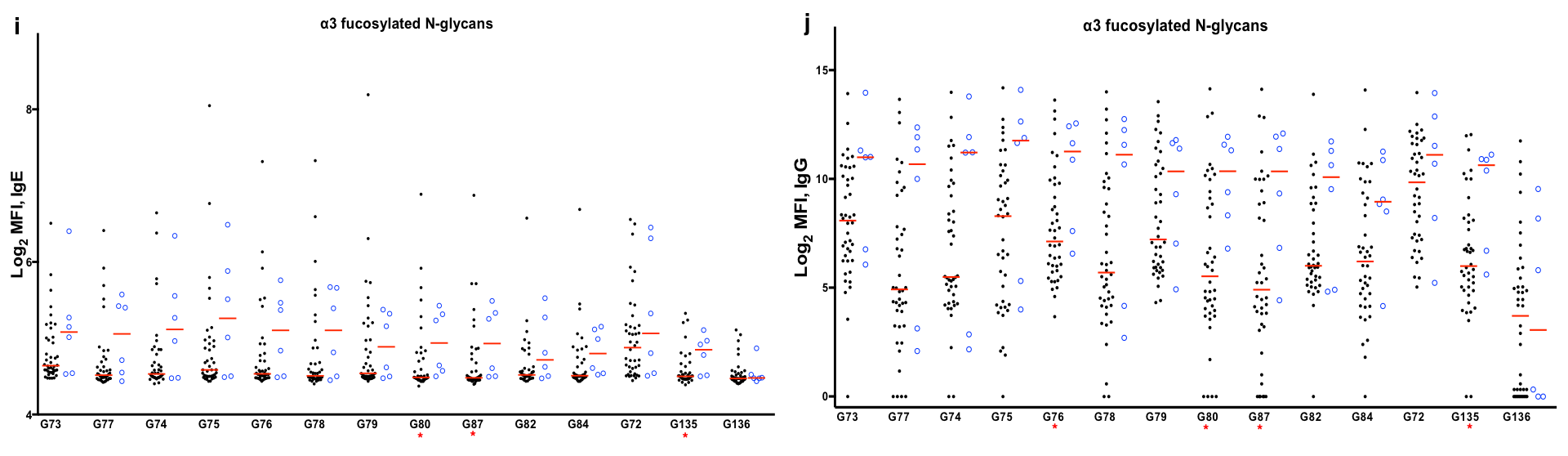**  **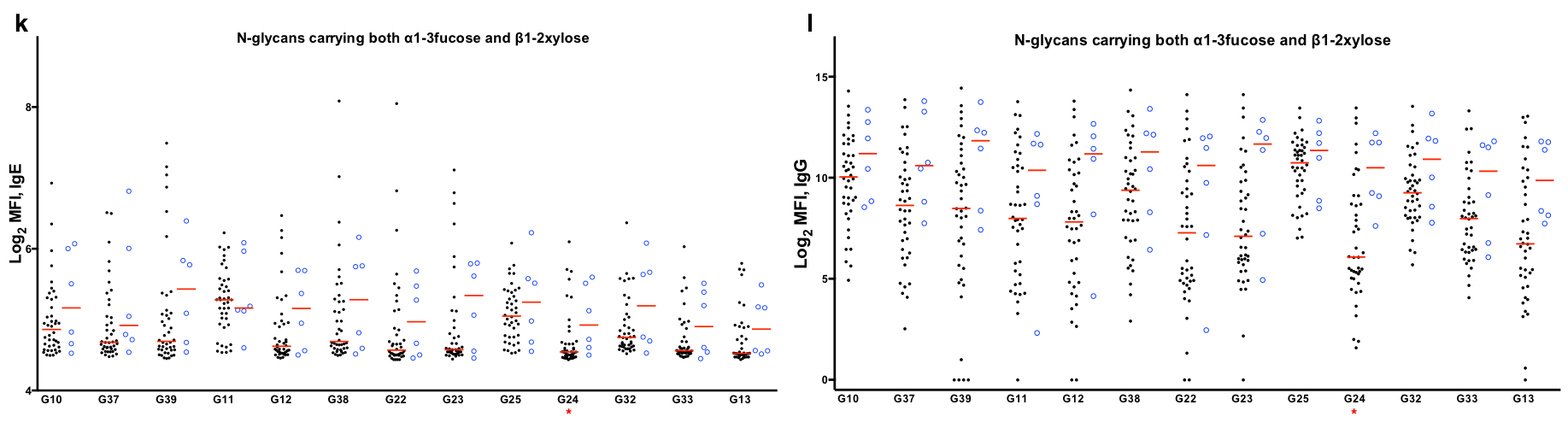**  **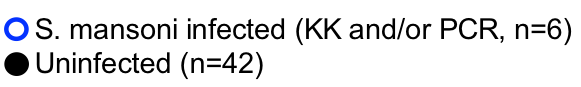** |
| *Figure shows background-subtracted and log_2_-transformed median fluorescence intensities (MFIs) representing IgE (a, c, e, g, i, k) and IgG (b, d, f, h, j, l) reactivity to α-1,3-fucosylated and β-1,2-xylosylated N-glycans, among S. mansoni infected (KK and/or PCR) [open circles] and uninfected (closed circles) rural and urban individuals. Mann-Whitney (IgE responses) and unpaired t test (IgG responses) were conducted within the framework of a Monte Carlo simulation algorithm based on 1000 permutations, to assess differences between infected and uninfected individuals.*  ********p<0.05;* *********p<0.01;* **********p<0.001* |

**Figure S3.** Associations between *S. mansoni* infection and IgE and IgG reactivity to N-glycans carrying GalNAcβ1-4GlcNAc (LDN), GalNAcβ1-4(Fucα1-3)GlcNAc (LDNF) and Galβ1-4(Fucα1-3)GlcNAc (LeX) antennae

| **RURAL** |
| --- |
| **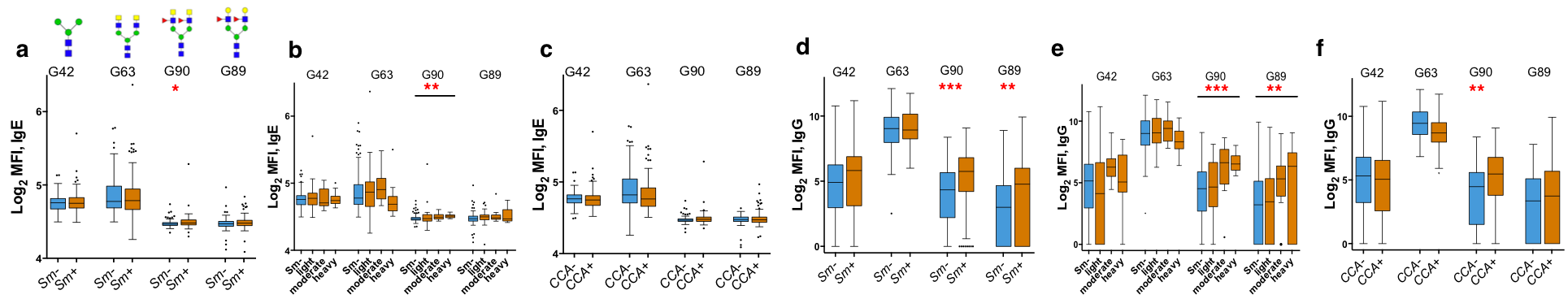** |
| **URBAN** |
| **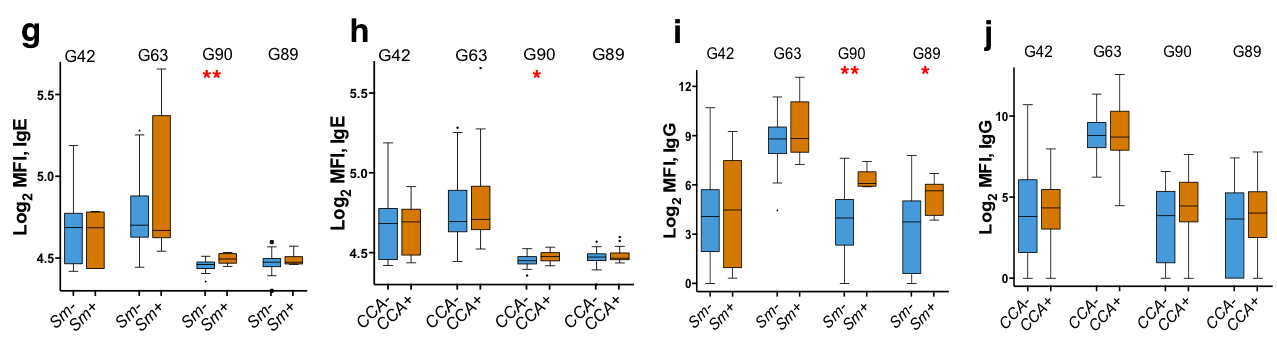** |
| *Plasma from S. mansoni infected and uninfected rural and urban individuals were assessed for IgE and IgG reactivity to N-glycan structural variants. Box-and-whisker plots show background-subtracted and log_2_-transformed median fluorescence intensities (MFI) representing IgE (a, b, c, g, h) and IgG (d, e, f, i, j) reactivity to the conserved, non-modified N-glycan core structure (Man_3_GlcNAc_2_,* ***G42****) and to N-glycan core structures carrying LDN (****G63****), LDNF (****G90****) and Lewis X (****G89****) antennae.*  *Mann-Whitney (IgE responses) and unpaired t test (IgG responses) were conducted within the framework of a Monte Carlo simulation algorithm based on 1000 permutations, to assess differences between infected and uninfected individuals. The Kruskal-Wallis (IgE responses) and one-way ANOVA test (IgG responses) were also conducted using the permutation approach to assess differences along the infection intensity gradient (b and e) in the rural survey.*  ********p<0.05;* *********p<0.01;* **********p<0.001.*  ***Sm:*** *S. mansoni infection determined by detection of eggs in a single stool sample by Kato-Katz and/or PCR (rural infected n=84, uninfected n=113; urban infected n=6, uninfected n=42).*  ***CCA:*** *S. mansoni infection determined by a positive urine circulating cathodic antigen (CCA) result (rural infected n=118, uninfected n=81; urban infected n=21, uninfected n=37).* |

**Figure S4.** Hierarchical cluster analysis of anti-glycan IgE and IgG responses

| 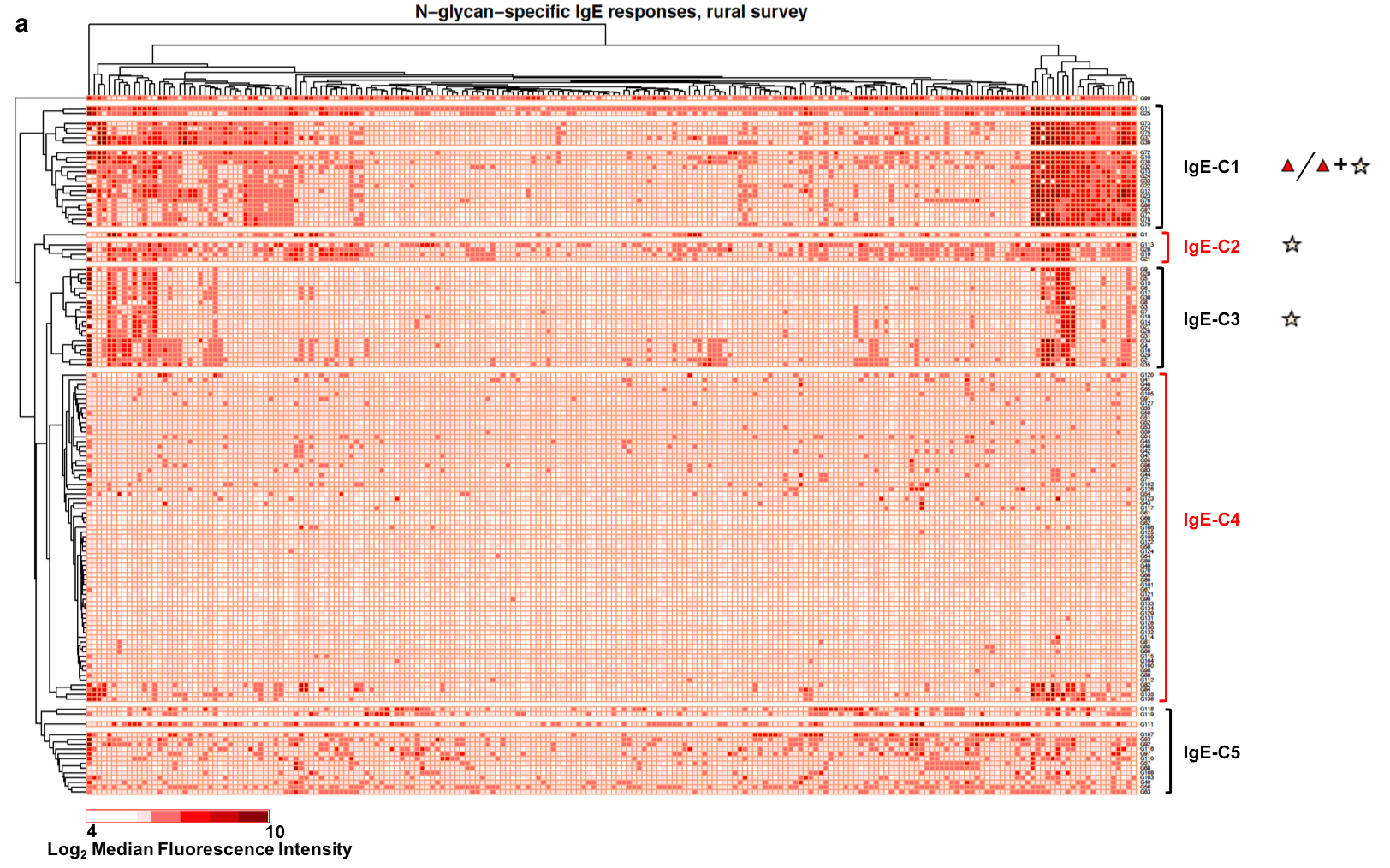 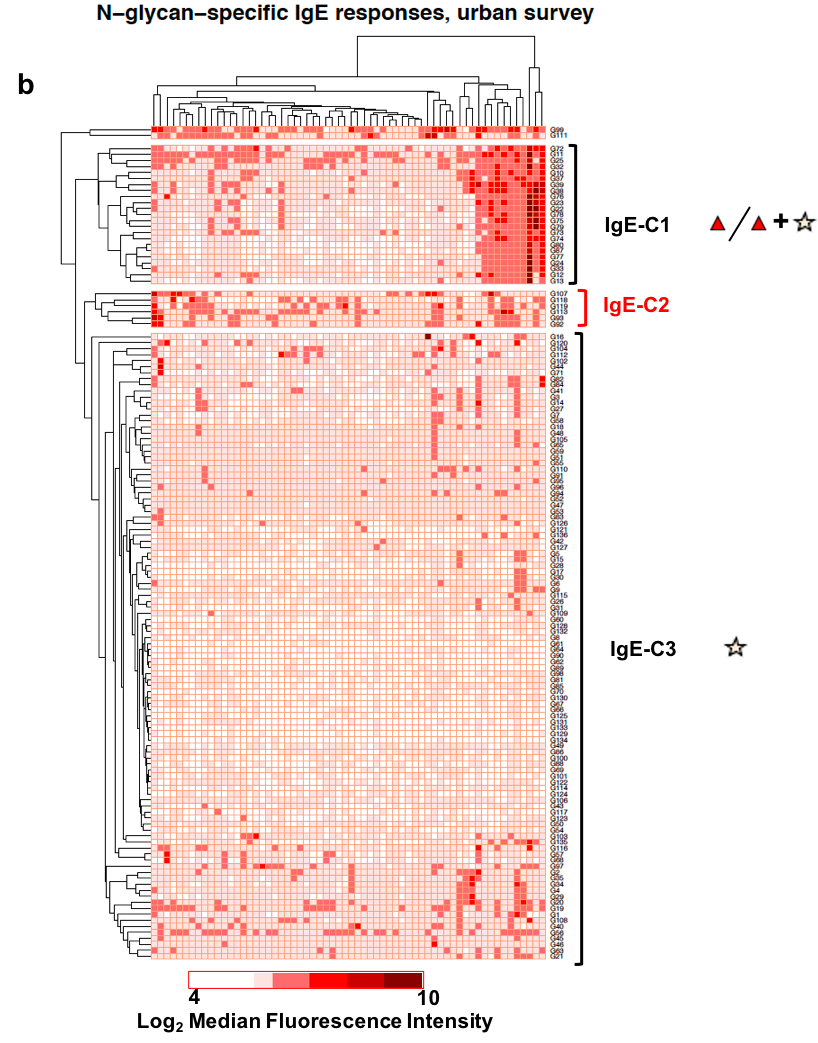  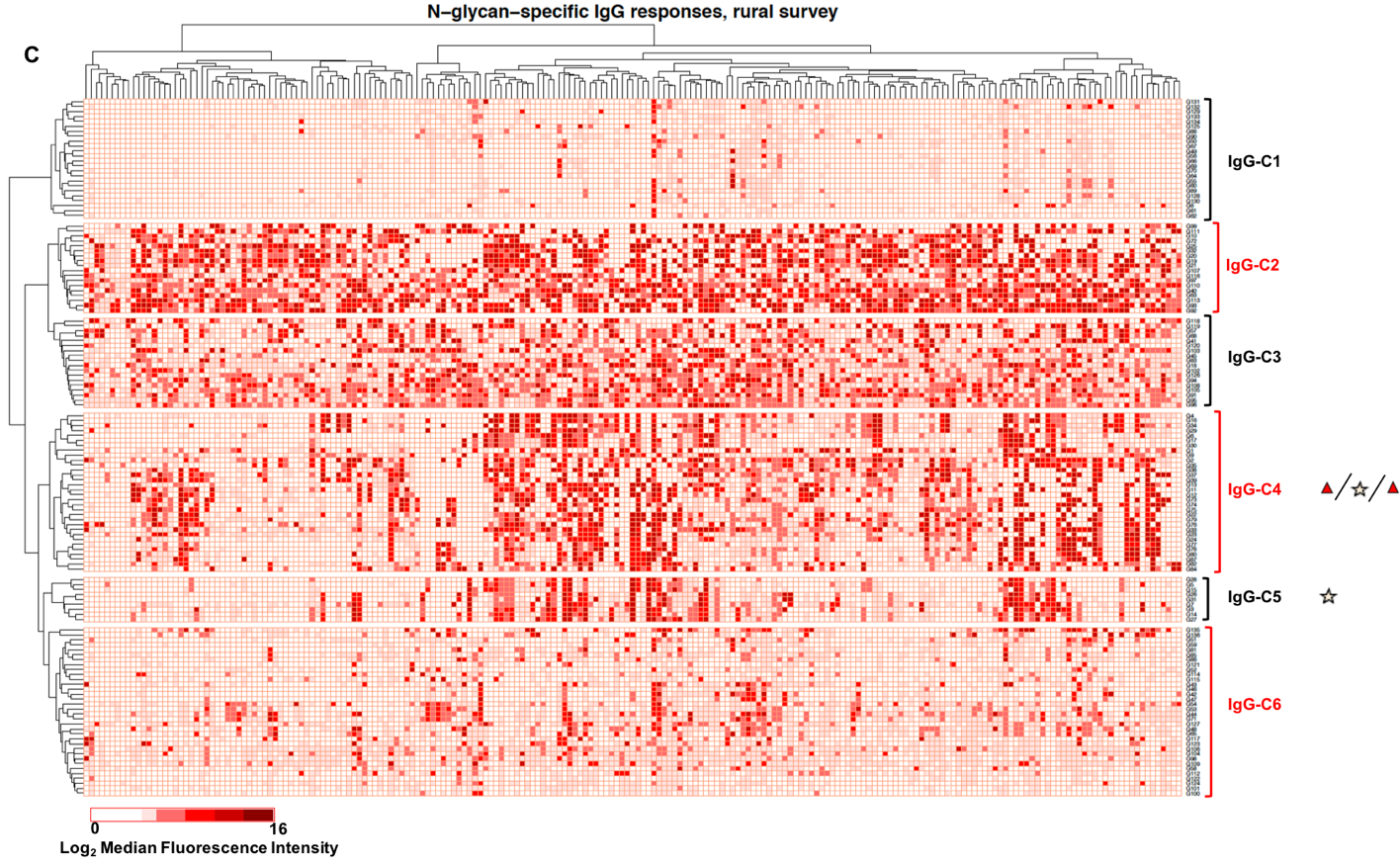 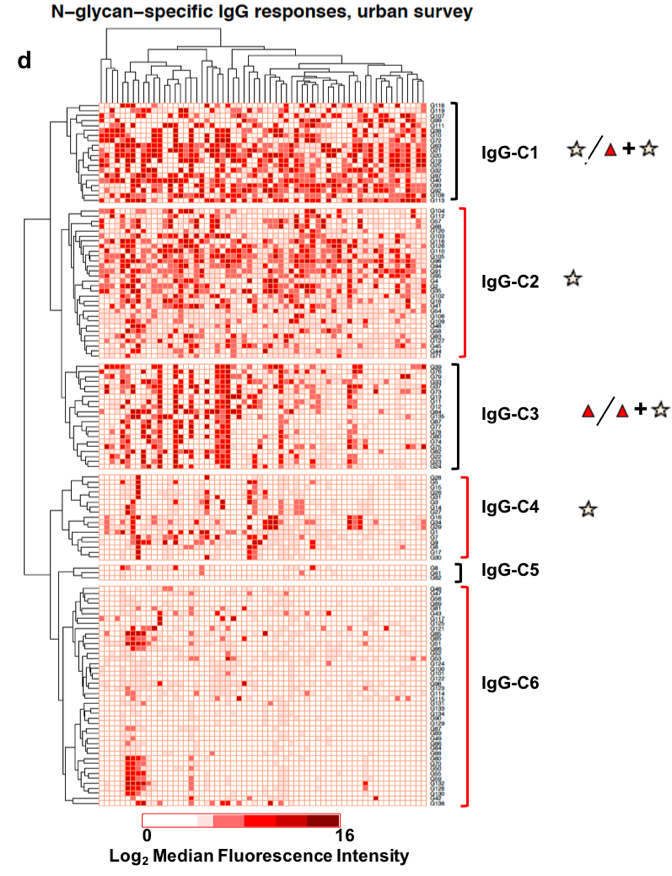 |
| --- |
| *Hierarchical cluster analysis (complete linkage using Euclidean distance) was conducted using the ‘pheatmap’ package in R. Background-subtracted and log_2_-transformed IgE and IgG median fluorescence intensities are shown on the y-axes, within clusters. The dominant core modifications on the glycans in these clusters are also shown. X-axes represent individual participants.* ***a)*** *Five major clusters of IgE responses were identified in the rural survey: cluster 1 (IgE-C1) comprised glycans carrying either only the core α3Fuc or both the core α3Fuc and β2Xyl, IgE-C2 and IgE-C3 comprised β-1,2-xylosylated glycans only, IgE-C4 and IgE-C5 comprised non-xylosylated and non-fucosylated glycans.* ***b)*** *Three major IgE clusters identified in the urban survey: IgE-C1 comprised glycans with core α3Fuc or both core α3Fuc and β2Xyl, IgE-C2 comprised non-xylosylated and non-fucosylated glycans, IgE-C3 comprised a mixture of β-1,2-xylosylated, non-xylosylated and non-fucosylated glycans.* ***c)*** *Six major IgG clusters identified in the rural survey: IgG-C1, IgG-C2, IgG-C3 and IgG-C6 were dominated by non-xylosylated and non-fucosylated glycans, IgG-C4 comprised glycans with either core α3Fuc or β2Xyl or both, IgG-C5 comprised β-1,2-xylosylated glycans only.* ***d)*** *Six major IgG clusters in the urban survey: IgG-C1 comprised glycans with either only core β2Xyl or both the core α3Fuc and β2Xyl, IgG-C2 a mixture of β-1,2-xylosylated, non-xylosylated and non-fucosylated glycans, IgG-C3 comprised glycans carrying either only core α3Fuc or both core α3Fuc and β2Xyl. IgE-C5 and IgE-C6 comprised non-xylosylated and non-fucosylated glycans.* |

**Table S2.** Global test p-values for associations between anti-glycan antibody response clusters and *S. mansoni* infection

| **RURAL** | IgE-C1  (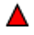 / 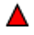 + 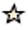) | IgE-C2  (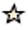) | IgE-C3  (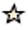) | IgE-C4 | IgE-C5 |  |  |
| --- | --- | --- | --- | --- | --- | --- | --- |
|  |  |  |  |  |  |  |  |
| *Sm* (KK) | 0.284 | **<0.001** | **0.019** | **0.036** | 0.533 |  |  |
| *Sm* (PCR) | 0.795 | **0.039** | 0.353 | 0.431 | 0.556 |  |  |
| CCA | 0.872 | 0.681 | 0.617 | 0.928 | 0.992 |  |  |
| Infection intensity (KK) | 0.385 | **<0.001** | **0.003** | **0.032** | 0.850 |  |  |
|  |  |  |  |  |  |  |  |
| **URBAN** | IgE-C1  (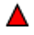 / 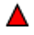 + 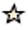) | IgE-C2 | IgE-C3  (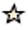) |  |  |  |  |
|  |  |  |  |  |  |  |  |
| *Sm* (KK) | 0.371 | 0.826 | **0.002** |  |  |  |  |
| *Sm* (PCR) | 0.314 | 0.604 | 0.134 |  |  |  |  |
| CCA | 0.733 | 0.652 | 0.421 |  |  |  |  |
| Infection intensity (KK) | 0.904 | 0.925 | **<0.001** |  |  |  |  |
|  |  |  |  |  |  |  |  |
|  |  |  |  |  |  |  |  |
| **RURAL** | IgG-C1 | IgG-C2 | IgG-C3 | IgG-C4  (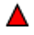 / 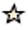 / 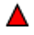) | IgG-C5  (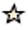) | IgG-C6 |  |
|  |  |  |  |  |  |  |  |
| *Sm* (KK) | 0.319 | **0.028** | 0.150 | 0.064 | 0.056 | 0.451 |  |
| *Sm* (PCR) | 0.701 | 0.373 | 0.241 | 0.180 | 0.090 | 0.539 |  |
| CCA | 0.175 | 0.934 | 0.939 | 0.695 | 0.678 | 0.394 |  |
| Infection intensity (KK) | 0.117 | 0.054 | 0.196 | **0.019** | **0.024** | 0.285 |  |
|  |  |  |  |  |  |  |  |
| **URBAN** | IgG-C1  (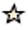 / 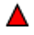 + 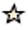) | IgG-C2  (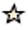) | IgG-C3  (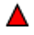 / 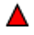 + 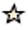) | IgG-C4  (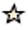) | IgG-C5 | IgG-C6 |  |
|  |  |  |  |  |  |  |  |
| *Sm* (KK) | 0.834 | 0.077 | 0.371 | 0.066 | **0.031** | 0.237 |  |
| *Sm* (PCR) | 0.498 | 0.252 | 0.180 | 0.103 | 0.569 | 0.408 |  |
| CCA | 0.775 | 0.875 | 0.945 | 0.253 | 0.596 | 0.679 |  |
| Infection intensity (KK) | 0.807 | 0.167 | 0.828 | 0.106 | **0.022** | 0.368 |  |
| *Table shows age and sex-adjusted global test p-values obtained from a linear regression analysis in R using the ‘Globaltest’ package. The global test, described by Goeman et al., 2006*[*^1^*](#_ENREF_1)*, was used here to assess associations between anti-glycan IgE and IgG clusters (from hierarchical cluster analysis) and S. mansoni infection. All statistically significant associations shown in this table are positive, except for the association between IgG-C5 (Urban survey) and Sm (KK) and infection intensity.*  ***KK****: Kato-Katz;* ***PCR****: Polymerase Chain Reaction;* ***CCA****: Circulating Cathodic Antigen; Sm: Schistosoma mansoni* | | | | | | | |

**Table S3.** Global test p-values for associations between anti-glycan antibody response clusters and *Schistosoma-*specific antibodies

| **RURAL** | IgE-C1  (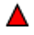 / 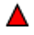 + 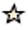) | IgE-C2  (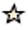) | IgE-C3  (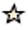) | IgE-C4 | IgE-C5 |  |  |
| --- | --- | --- | --- | --- | --- | --- | --- |
|  |  |  |  |  |  |  |  |
| SWA-specific IgE | **<0.001** | **0.003** | **0.001** | **0.008** | 0.073 |  |  |
| SEA-specific IgE | **<0.001** | **0.008** | **<0.001** | 0.523 | **0.031** |  |  |
|  |  |  |  |  |  |  |  |
| **URBAN** | IgE-C1  (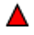 / 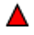 + 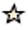) | IgE-C2 | IgE-C3  (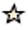) |  |  |  |  |
|  |  |  |  |  |  |  |  |
| SWA-specific IgE | **<0.001** | 0.255 | **<0.001** |  |  |  |  |
| SEA-specific IgE | **0.021** | 0.372 | **0.022** |  |  |  |  |
|  |  |  |  |  |  |  |  |
| **RURAL** | IgG-C1 | IgG-C2 | IgG-C3 | IgG-C4  (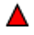 / 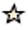 / 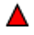) | IgG-C5  (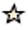) | IgG-C6 |  |
|  |  |  |  |  |  |  |  |
| SWA-specific IgG | 0.079 | **<0.001** | **0.005** | **<0.001** | **<0.001** | 0.077 |  |
| SEA-specific IgG | 0.453 | **<0.001** | **0.012** | **<0.001** | **0.003** | 0.221 |  |
|  |  |  |  |  |  |  |  |
| **URBAN** | IgG-C1  (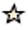 / 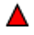 + 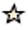) | IgG-C2  (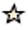) | IgG-C3  (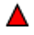 / 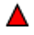 + 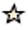) | IgG-C4  (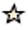) | IgG-C5 | IgG-C6 |  |
|  |  |  |  |  |  |  |  |
| SWA-specific IgG | 0.219 | 0.518 | **0.034** | **0.045** | 0.872 | 0.357 |  |
| SEA-specific IgG | 0.063 | 0.303 | **0.016** | 0.075 | 0.918 | 0.434 |  |
| *Table shows age and sex-adjusted global test p-values obtained from a linear regression analysis in R using the ‘Globaltest’ package. The global test (Goeman et al., 2006*[*^1^*](#_ENREF_1)*) was used to assess associations between anti-glycan IgE and IgG clusters (defined by hierarchical clustering analysis) and Schistosoma adult worm (SWA) and egg (SEA)-specific IgE and IgG. All statistically significant associations shown in this table are positive.* | | | | | | | |

**Figure S5.** Hierarchical cluster analysis of anti-glycan antibody responses in individuals from both rural and urban settings

| 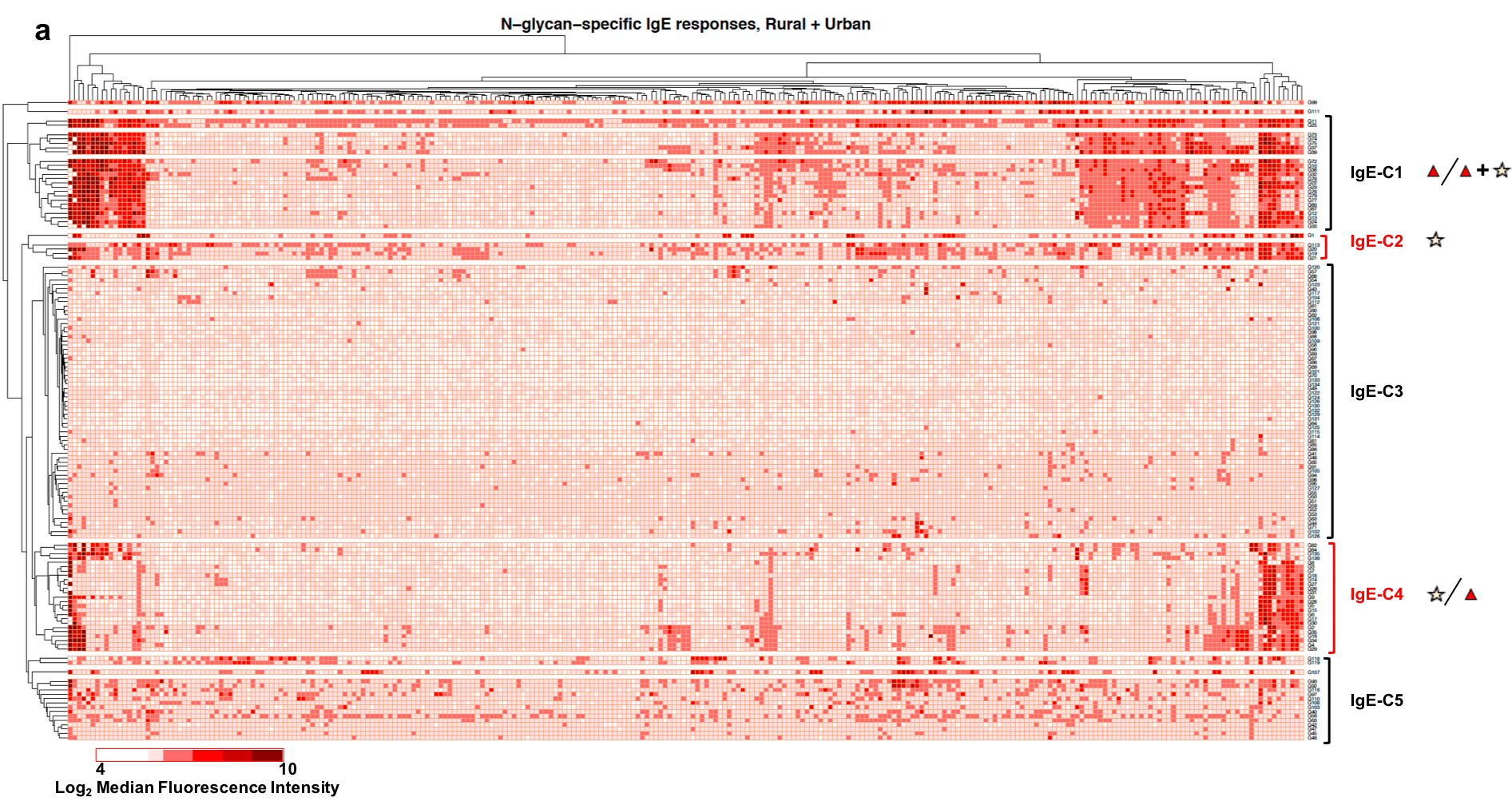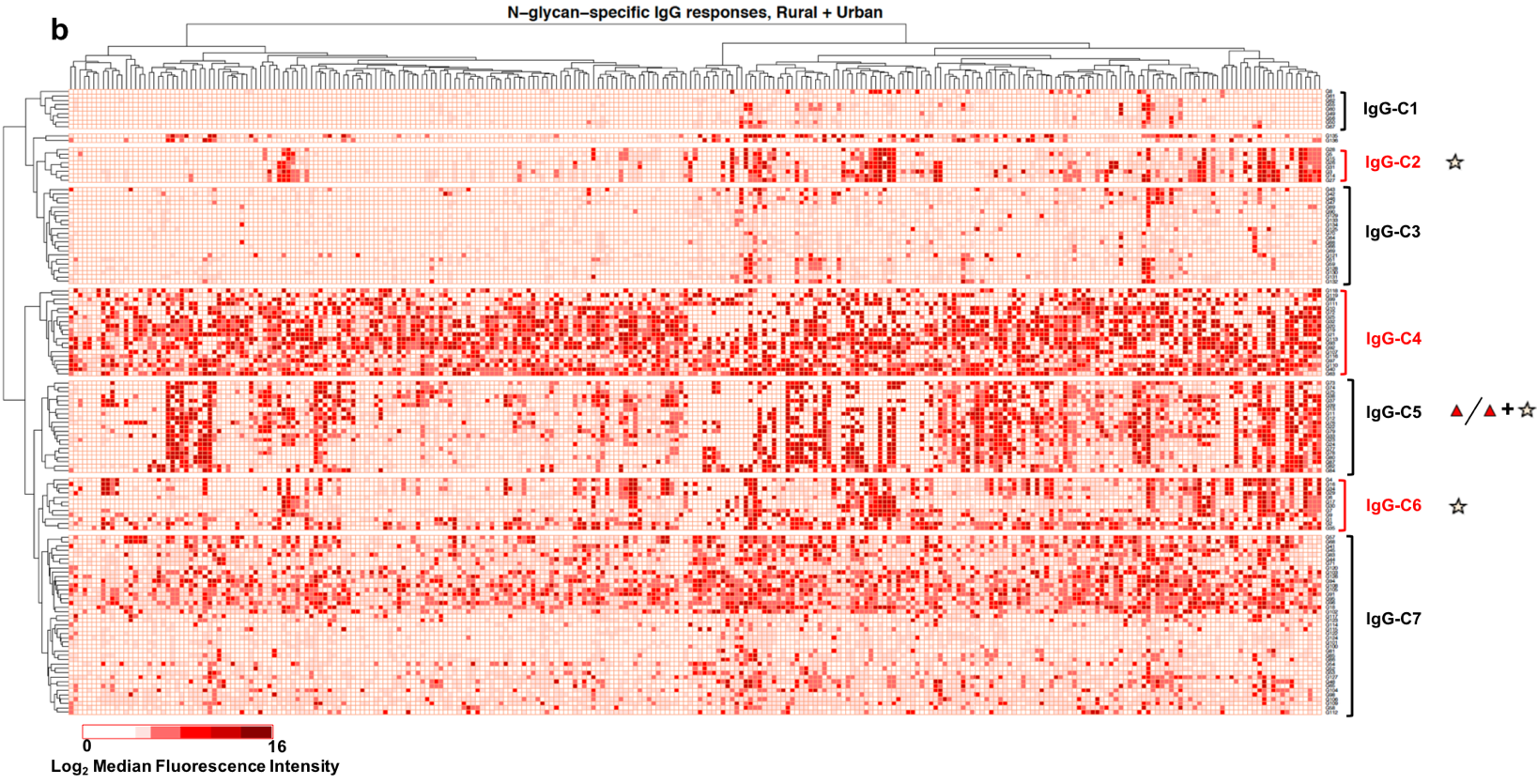 |
| --- |
|  |
| *Hierarchical cluster analysis (complete linkage using Euclidean distance) of N-glycan-specific IgE and IgG responses (y-axis). X-axes represent individual participants.* |

**Table S4.** Global test p-values for associations between anti-glycan antibody response clusters and survey setting

| IgE-C1  (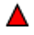/ 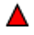 + 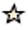) | IgE-C2  (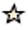) | IgE-C3 | IgE-C4  (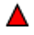 / 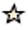) | IgE-C5 |  |  |
| --- | --- | --- | --- | --- | --- | --- |
|  |  |  |  |  |  |  |
| 0.095* | **0.024*** | **0.006^#^** | 0.094* | 0.146* |  |  |
|  |  |  |  |  |  |  |
| IgG-C1 | IgG-C2  (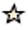) | IgG-C3 | IgG-C4 | IgG-C5  (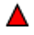/ 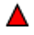 + 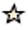) | IgG-C6  (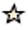) | IgG-C7 |
|  |  |  |  |  |  |  |
| 0.245^#^ | 0.508* | 0.179* | 0.222* | 0.123* | 0.223* | **0.009^#^** |
| *Table shows age and sex-adjusted global test p-values obtained from a linear regression analysis in R using the ‘Globaltest’ package. The global test (Goeman et al., 2006*[*^1^*](#_ENREF_1)*) was used to assess associations between anti-glycan IgE and IgG clusters (defined by hierarchical clustering analysis) and survey setting.*  ********Positive association between antibody response cluster and rural setting*  ***^#^****Positive association between antibody response cluster and urban setting* | | | | | | |

**REFERENCES**

1 Goeman, J. J., Van De Geer, S. A. & Van Houwelingen, H. C. Testing against a high dimensional alternative. *Journal of the Royal Statistical Society: Series B* **68**, 477-493 (2006).
